# Maintenance DNA methylation is required for induced Treg reparative function following viral pneumonia in mice

**DOI:** 10.1172/JCI192925

**Published:** 2025-09-16

**Authors:** Anthony M. Joudi, Jonathan K. Gurkan, Qianli Liu, Elizabeth M. Steinert, Manuel A. Torres Acosta, Kathryn A. Helmin, Luisa Morales-Nebreda, Nurbek Mambetsariev, Carla Patricia Reyes Flores, Hiam Abdala-Valencia, Samuel E. Weinberg, Benjamin D. Singer

**Affiliations:** 1 Division of Pulmonary and Critical Care Medicine,; 2 Medical Scientist Training Program,; 3 Driskill Graduate Program,; 4 Division of Allergy and Immunology,; 5 Department of Pathology,; 6 Department of Biochemistry and Molecular Genetics,; 7 Simpson Querrey Institute for Epigenetics, and; 8 Simpson Querrey Lung Institute for Translational Science (SQ LIFTS), Northwestern University Feinberg School of Medicine, Chicago, Illinois, USA.

**Keywords:** Immunology, Inflammation, Pulmonology, Epigenetics, Influenza, T cells

## Abstract

FOXP3^+^ natural regulatory T cells (nTregs) promote resolution of inflammation and repair of epithelial damage following viral pneumonia–induced lung injury, thus representing a cellular therapy for patients with severe viral pneumonia and the acute respiratory distress syndrome. Whether in vitro–induced Tregs (iTregs), which can be rapidly generated in substantial numbers from conventional T cells, also promote lung recovery is unknown. nTregs require specific DNA methylation patterns maintained by the epigenetic regulator ubiquitin-like with PHD and RING finger domains 1 (UHRF1). Here, we tested whether iTregs promote recovery following viral pneumonia and whether iTregs require UHRF1 for their pro-recovery function. We found that adoptive transfer of iTregs to mice with influenza virus pneumonia promotes lung recovery and that loss of UHRF1-mediated maintenance DNA methylation in iTregs leads to reduced engraftment and a delayed repair response. Transcriptional and DNA methylation profiling of adoptively transferred UHRF1-deficient iTregs that had trafficked to influenza-injured lungs demonstrated transcriptional instability with gain of transcription factors that define effector T cell lineage. Strategies to promote the stability of iTregs could be leveraged to further augment their pro-recovery function during viral pneumonia and other causes of severe lung injury.

## Abstract 2

We gain mechanistic insights into how maintenance DNA methylation stabilizes induced regulatory T cells to promote repair of the alveolar epithelium during viral pneumonia.

## Graphical abstract

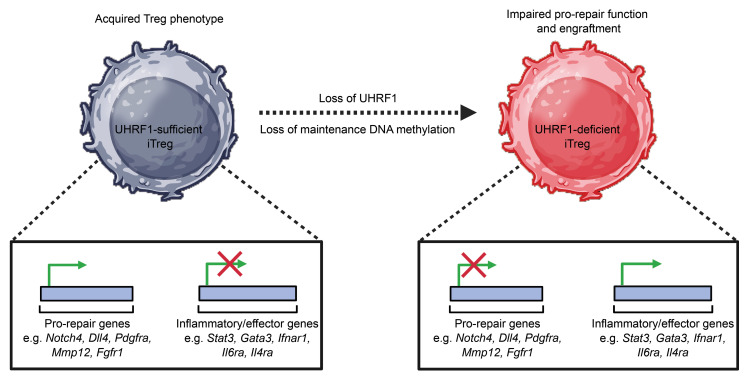

## Introduction

Regulatory T cells (Tregs) are a subset of CD4^+^ T cells that prevent spontaneous autoimmunity and mitigate exuberant immune responses by promoting self-tolerance and anergy (1, 2). Tregs also promote repair in multiple tissue types, including the lungs, following acute injury due to infection or sterile triggers (3–11). To maintain their identity and function, Tregs require expression of the lineage-determining transcription factor FOXP3, and maintenance of a signature DNA methylation landscape at key genomic elements, such as the *Foxp3* super-enhancer and other Treg-specific super-enhancers (Treg-SE) (12–14). Natural regulatory T cells (nTregs) emigrate from the thymus expressing FOXP3 and with the Treg lineage–determining DNA methylation landscape in place (12, 13). These cells are relatively rare — 5%–15% of circulating CD4^+^ T cells — and, unfortunately, are difficult to expand ex vivo, posing a barrier to therapeutic Treg transfer protocols (15). Induced regulatory T cells (iTregs) derive from conventional (FOXP3^–^) CD4^+^ T (Tconv) cells that express FOXP3 after culture in the presence of TGF-β, IL-2, and T cell receptor stimulation, resulting in gain of nTreg-like suppressive functions. As iTregs are derived in vitro from CD4^+^ Tonv cells, a more abundant cell type, they can expand more robustly and rapidly than nTregs, an attractive feature for clinical use. Whether iTregs function like nTregs to promote recovery following acute lung injury is not known. Moreover, iTregs do not carry the signature DNA methylation landscape seen in nTregs, leading to transcriptional instability in inflammatory microenvironments and potential conversion to proinflammatory T cell phenotypes (12, 16–18). Indeed, modulation of the epigenetic landscape via DNA methyltransferase inhibition or TET enzyme activation augments the stability and function of natural and induced Tregs (8, 19–25). Elucidating targetable mechanisms that stabilize iTreg transcriptional programs and function therefore represents an important objective for developing Treg-based therapies that promote recovery from severe pneumonia (15, 26).

The epigenetic regulator ubiquitin-like with PHD and RING finger domains 1 (UHRF1; also known as Np95 in mice and ICBP90 in humans) is a multidomain non-redundant adaptor protein that recruits the maintenance DNA methyltransferase DNMT1 to replicating daughter DNA strands to maintain cell type–specific methylation patterns during DNA replication (27–29). UHRF1 is required for the stability of nTreg identity; loss of UHRF1 in nTregs during thymic development or in the adult mouse leads to generation of inflammatory ex-FOXP3 (i.e., ex-Treg) cells (30). In contrast, the necessity of UHRF1 in regulating iTreg stability and function is less clear. Published data suggest that UHRF1 is dispensable for iTreg generation (30, 31), yet others have reported that iTreg generation from UHRF1-deficient CD4^+^ Tconv cells augments their suppressive function in a colitis model of inflammation (31). We hypothesized that iTregs require UHRF1-mediated maintenance DNA methylation to stabilize their acquired transcriptional and functional programs.

To test our hypothesis, we performed adoptive transfer of UHRF1-sufficient or -deficient iTregs into Treg-depleted mice with influenza A virus pneumonia, which do not recover from lung injury in the absence of reconstitution of the Treg population. Adoptive transfer of UHRF1-sufficient iTregs promoted recovery similarly to adoptive transfer of nTregs. In contrast, we found that recipients of UHRF1-deficient iTregs suffered worsened hypoxemia and mortality as well as delayed alveolar epithelial repair compared with mice that received UHRF1-sufficient iTregs. UHRF1-deficient iTregs displayed reduced lung engraftment at early and late recovery time points. Loss of UHRF1-mediated maintenance DNA methylation had no effect on FOXP3 induction yet resulted in a significant instability in vivo and caused significant transcriptomic instability at other core Treg loci in vitro and in vivo during viral pneumonia. In summary, UHRF1-mediated maintenance DNA methylation stabilizes iTreg cellular identity and reparative function following viral pneumonia.

## Results

### nTregs and iTregs promote recovery following viral pneumonia.

Transient depletion of FOXP3^+^ nTregs is followed by a renewal of the FOXP3^+^ population (32). To determine when after viral infection FOXP3^+^ Tregs promote recovery and optimize the timing of Treg adoptive transfer, we first assessed whether the timing of Treg renewal determines influenza pneumonia recovery phenotypes. To deplete Tregs, we administered loading doses of diphtheria toxin (DTx) to *Foxp3^GFP-DTR^* mice 2 days before sublethal influenza A virus infection and continued DTx administration every 2 days until 6, 10, 14, or 21 days post-infection (DPI) (Supplemental Figure 1A; supplemental material available online with this article; https://doi.org/10.1172/JCI192925DS1). We previously reported that DTx administration to wild-type mice with viral pneumonia does not contribute to immunopathology (33). Here, we confirmed depletion of Tregs in the spleen at 6 DPI (4 doses of DTx) in *Foxp3^GFP-DTR^* mice (Supplemental Figure 1B). *Foxp3^GFP-DTR^* mice that received PBS and *Foxp3^Cre^* mice that received DTx were included for comparison and demonstrated a typical frequency of endogenous Tregs in the spleen (Supplemental Figure 1B). An analysis of mice at 13 DPI that had DTx withdrawn at 6 DPI revealed that the Treg population in the spleen and lung was renewing, but had not yet returned to baseline, in comparison with infected mice that had not received DTx (Supplemental Figure 1C). Following influenza infection, we found that *Foxp3^GFP-DTR^* mice that had DTx withdrawn at 6 DPI recovered their mass faster than mice that continued to receive DTx through 10, 14, or 21 DPI (Supplemental Figure 1D). Because administration of DTx alone results in some loss of mass (34), and thus this measure may also reflect DTx withdrawal timing rather than degree of resolving lung injury, we confirmed and compared the degree of lung injury in representative mice via histology at 60 DPI. While all groups had evidence of residual lung injury, the quantification of damaged lung tissue revealed it was most severe in mice that continued to receive DTx through 21 DPI (Supplemental Figure 1E). In response to antigen or inflammation in vivo, some (FOXP3^–^) CD4^+^ Tconv cells transiently express FOXP3 but not the signature DNA methylation pattern characteristic of nTregs; these cells are known as peripheral Tregs (35–39). Accordingly, we determined the DNA methylation profile of FOXP3^+^ cells that repopulate following withdrawal of DTx in the influenza virus pneumonia model. Genome-wide DNA methylation profiling revealed that the Treg-SE DNA methylation profile of the renewed FOXP3^+^ population aligned with an nTreg-type profile when compared with direct ex vivo naive splenic nTregs and iTregs harvested on day 5 of culture (Supplemental Figure 1F).

**Figure 1:**
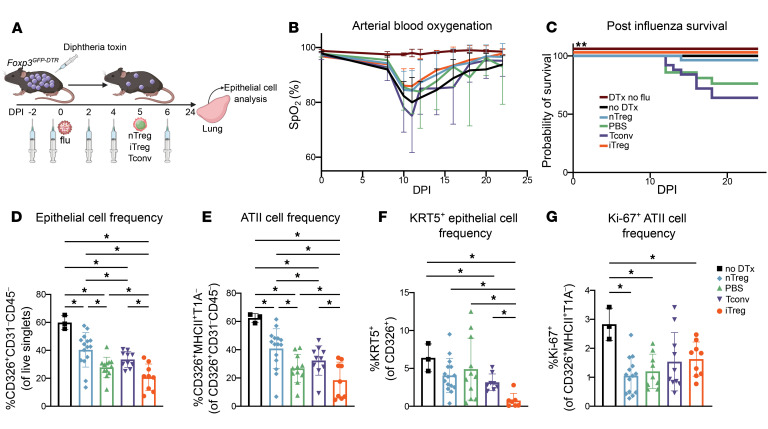
Adoptive transfer of iTregs promotes survival following viral pneumonia. *Foxp3^GFP-DTR^* mice were treated with DTx every 48 hours beginning 2 days before inoculation with 6.5 PFU of influenza A/WSN/33 H1N1 virus and at 5 DPI received retro-orbital adoptive transfer of 1 × 10^6^ nTregs, iTregs, or Tconv cells or PBS. Additional controls received DTx but no influenza inoculation (DTx no flu) or influenza inoculation but no diphtheria toxin (no DTx). (**A**) Schematic of experimental design. (**B**) Mice were followed over time for arterial oxyhemoglobin saturation (SpO_2_) measured via dorsal collar clip. (**C**) Survival of mice that received indicated treatments. (**D**–**G**) Mice were euthanized at 24 DPI, and lungs were analyzed by flow cytometry for frequency of epithelial, CD326^+^CD31^–^CD45^–^ cells (**D**); frequency of ATII, CD326^+^MHCII^+^T1A^–^ cells (**E**); frequency of KRT5^+^CD326^+^ epithelial cells (**F**); and frequency of Ki-67^+^CD326^+^MHCII^+^T1A^–^ cells (**G**). Data from recipients of iTregs are derived from post-caval lobe; data from all other groups are derived from whole-lung suspensions. (**B**, DTx no flu *n* = 4, no DTx *n* = 6, nTreg *n* = 11, PBS *n* = 9, Tconv *n* = 12, iTreg *n* = 18; **C**, DTx no flu *n* = 4, no DTx *n* = 9, nTreg *n* = 27, PBS *n* = 21, Tconv *n* = 25, iTreg *n* = 18; **D** and **E**, no DTx *n* = 3, nTreg *n* = 15, PBS *n* = 11, Tconv *n* = 10, iTreg *n* = 9; **F**, no DTx *n* = 3, nTreg *n* = 15, PBS *n* = 11, Tconv *n* = 8, iTreg *n* = 7; **G**, no DTx *n* = 3, nTreg *n* = 14, PBS *n* = 9, Tconv *n* = 10, iTreg *n* = 9). Data in **B** generated from 4 independent experiments. Data in **C** generated from 5 independent experiments. Data in **D**–**G** generated from 4 independent experiments. Survival curve (**C**) *P* value was determined using log-rank (Mantel-Cox) test, **P < 0.005. Data presented as mean and SD with *q < 0.05 according to multiple Mann-Whitney tests and correcting for multiple comparisons using the 2-stage linear step-up procedure of Benjamini, Krieger, and Yekutieli with *Q* = 5% (**D**–**G**).

To generate lineage-identifiable iTregs for adoptive transfer, we first bred mice harboring a tamoxifen-inducible *Foxp3*-Cre driver with a green fluorescent protein (GFP) label (*Foxp3^GFP-CreERT2^*) and a *loxP*-flanked stop codon upstream of the red fluorescent protein, tdTomato, driven by a CAG promoter at the open *Rosa26* locus (*Rosa26Sor^CAG-tdTomato^*) (30). Then, sorted CD4^+^ Tconv cells (CD4^+^*Foxp3*-GFP^–^) from the secondary lymphoid organs of *Foxp3^GFP-CreERT2^Rosa26Sor^CAG-tdTomato^* mice were cultured in the presence of T cell receptor stimulation (αCD3ε/αCD28), TGF-β, IL-2, and tamoxifen to induce FOXP3^+^ cell–specific GFP and tdTomato expression (Supplemental Figure 2A). Separately, CD4^+^ Tconv cells derived from the same mice were cultured in the presence of T cell receptor stimulation and IL-2, to serve as controls (Supplemental Figure 2A). iTregs and Tconv cells were harvested for adoptive transfer on day 5 of cell culture. nTregs were adoptively transferred directly after isolation from the spleen and lymph nodes of *Foxp3^GFP-CreERT2^Rosa26Sor^CAG-tdTomato^* mice (Supplemental Figure 2B). Concurrently, recipient *Foxp3^GFP-DTR^* mice received DTx followed by intratracheal instillation with a titer of influenza A virus sufficient to cause 10%–20% mortality in Treg-depleted animals. At 5 DPI, 1 × 10^6^ iTregs, nTregs, or Tconv cells or PBS were administered via retro-orbital injection (Figure 1A). Although arterial oxyhemoglobin saturation (SpO_2_) was similar between groups, mice that received influenza but no DTx (positive control), nTregs (positive control), or iTregs experienced significantly greater survival compared with mice that received Tconv cells (negative control) or PBS (vehicle control) (Figure 1, B and C). Mice that received influenza but no DTx displayed a more rapid recovery in mass as well as a significantly lower absolute number of lung-infiltrating leukocytes compared with the other groups (Supplemental Figure 2, C and D). Flow cytometric analysis of lung single-cell suspensions at 24 DPI revealed a greater percentage of alveolar epithelial cells, including alveolar epithelial type II (ATII) cells (CD326^+^MHCII^+^T1A^–^/CD326^+^CD31^–^CD45^–^) (40), in mice that received nTregs or no DTx compared with mice that received Tconv cells or PBS (Figure 1, D and E). Unexpectedly, mice that received iTregs displayed the lowest percentage of ATII cells among the experimental groups. To further characterize the effect of iTreg adoptive transfer on epithelial repair, we examined the KRT5^+^ epithelial cell population, a marker of dysregulated and incomplete repair (41–43). Mice that received iTregs displayed the lowest percentage of KRT5^+^ epithelial cells, suggesting effective repair despite the reduced percentage of ATII cells (Figure 1F). We observed no significant differences in the frequency of proliferating (Ki-67^+^) ATII cells between groups that received DTx (Figure 1G) or in absolute epithelial or endothelial cell numbers between groups (Supplemental Figure 2, E–I). To define and assess transcriptional signature differences between nTregs and iTregs after influenza infection, we compared the list of upregulated genes in iTregs harvested from lungs at 24 DPI with a previously defined gene cluster that is associated with repair function in nTregs harvested from the lungs of 8- to 12-week-old *Foxp3^GFP-DTR^* mice at a late repair time point (40). We found a high degree of similarity in gene expression between lung iTregs and nTregs during repair; this gene set included several genes known to be associated with Treg repair function (*Bmper*, *Ereg*, *Fgf1*, *Hhip*, *Hoxa5*, *Igf1*, *Lama3*, *Lox*, *Mmp12*, and *Pdgfa*) (Supplemental Figure 3A and Supplemental Table 1). Taken together, these results suggest that iTreg adoptive transfer is beneficial to alveolar epithelial repair.

### UHRF1 is dispensable for iTreg FOXP3 induction and stability but is required to maintain transcriptional and epigenetic programs in vitro.

**Figure 2:**
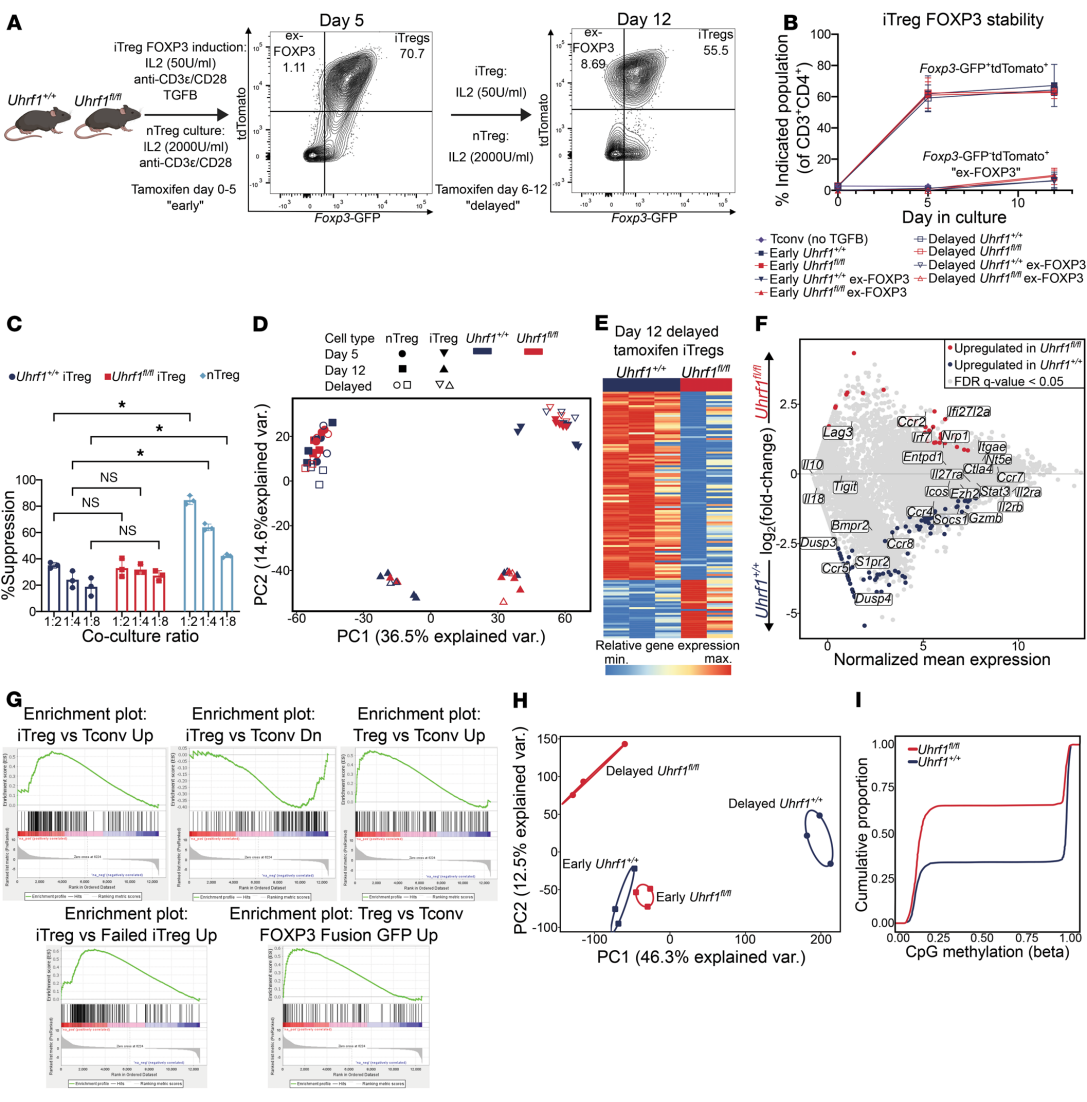
UHRF1 is dispensable for iTreg FOXP3 expression and suppressive capacity but is required for transcriptional and epigenetic stability in vitro. (**A**–**D**) Natural and induced Tregs derived from *Uhrf1^+/+^* and *Uhrf1^fl/fl^* mice were exposed to tamoxifen to delete UHRF1 from day 0 to day 5 (“early”) or day 6 to day 12 (“delayed”) of culture, then harvested on days 5 and 12 for flow cytometry and RNA-Seq analysis. (**A**) Schematic. (**B**) Frequency of *Foxp3*-GFP^+^tdTomato^+^ iTregs and *Foxp3*-GFP^–^tdTomato^+^ (ex-FOXP3) cells. (**C**) Percent suppression of CD4^+^CTV^+^*Foxp3*-GFP^–^ splenic responder T cells cocultured for 72 hours at indicated ratios of experimental Tregs. (**D**) PCA of 6,978 DEGs, identified from ANOVA-like testing with FDR *q* < 0.05. (**E**–**I**) Induced Tregs derived from *Uhrf1^+/+^* and *Uhrf1^fl/fl^* mice were exposed to tamoxifen to delete UHRF1 from day 6 to day 12 (“delayed”) of culture, then harvested on day 12 for RNA-Seq and DNA methylation analysis. (**E**) *K*-means clustering of 127 genes with an FDR *q* < 0.05 with *k* = 2. (**F**) MA plot comparing gene expression between groups. Genes of interest are annotated. (**G**) Enrichment plots of gene sets (*P* < 0.05, FDR *q* < 0.25) generated through GSEA pre-ranked testing of the expressed genes. (**H**) PCA of 81,179 differentially methylated cytosines identified from ANOVA-like testing with FDR *q* < 0.05. Ellipses represent normal contour lines with 1 SD probability. (**I**) Cumulative distribution function plot of differentially methylated cytosines expressed as β scores, with 0 representing unmethylated and 1 representing fully methylated; a shift in the cumulative distribution function up and to the left represents relative hypomethylation. (**B**, *n* = 3 per group; **C**, *n* = 3 per group; **D**–**I**, *n* = 2 for *Uhrf1^fl/fl^*, *n* = 3 for *Uhrf1^+/+^*). **B** and **C** representative of 3 independent biological replicates. *q < 0.05 according to 2-way ANOVA with 2-stage linear step-up procedure of Benjamini, Krieger, and Yekutieli with *Q* = 5 (**C**).

To test whether UHRF1-mediated maintenance DNA methylation is necessary for iTreg differentiation and stability in vitro, we first bred *Uhrf1^fl/fl^Foxp3^GFP-CreERT2^Rosa26Sor^CAG-tdTomato^* mice (referred to here as *Uhrf1^fl/fl^*). This gene combination results in inducible, Treg-specific, *Foxp3* lineage–traceable iTregs that lose UHRF1 expression contemporaneously with FOXP3 induction (30). CD4^+^*Foxp3*-GFP^+^ cells (nTregs) and CD4^+^*Foxp3*-GFP^–^ T cells were isolated from the spleens of *Uhrf1^+/+^* (control) or *Uhrf1^fl/fl^* mice. nTregs were cultured in the presence of αCD3ε/αCD28 activation beads at a ratio of 3 beads to 1 Treg and recombinant human IL-2 at a concentration of 2,000 U/mL. CD4^+^*Foxp3*-GFP^–^ T cells were cultured in αCD3ε/αCD28–coated plates, recombinant human IL-2 (50 U/mL), and TGF-β (10 ng/mL) (Figure 2A). From each genotype, one group of nTregs and iTregs was treated with tamoxifen during the initial 5 days of culture (denoted as the “early” group). Cells from this early culture were harvested and sorted for bulk RNA-Seq analysis on day 5 or cultured for an additional 7 days without tamoxifen and then sorted for bulk RNA-Seq analysis on culture day 12 (Figure 2A). A separate group of nTregs and iTregs that was not exposed to tamoxifen during the first 5 days of culture (denoted as the “delayed” group) was either sorted for bulk RNA-Seq on culture day 5 or transitioned into medium with tamoxifen and cultured for an additional 7 days before sorting for RNA-Seq analysis on culture day 12. Consistent with published data (30, 31), flow cytometry analysis demonstrated no significant difference in FOXP3 induction or stability in iTregs (i.e., ex-FOXP3 cells) over the culture period regardless of when UHRF1 was deleted (Figure 2B). We confirmed that CD4^+^*Foxp3*-GFP^+^ iTregs and nTregs from *Uhrf1^+/+^* mice sorted on day 5 of cell culture expressed high levels of canonical Treg signature genes (e.g., *Il2ra*, *Il2rb*, *Icos*, *Tigit*, *Il10*, *Gzmb*, *Ctla4*, *Nt5e*, *Itgae*, *Nrp1*, and *Lag3*) (Supplemental Figure 3, B and C). We additionally confirmed the in vitro suppressive function of iTregs following 5 days of culture, finding no significant difference between *Uhrf1^+/+^* and *Uhrf1^fl/fl^* cells (Figure 2C). Principal component analysis (PCA) of 6,978 differentially expressed genes (DEGs) identified from ANOVA-like testing with FDR *q* < 0.05 in “early” and “delayed” groups of *Uhrf1^+/+^* and *Uhrf1^fl/fl^* nTregs and iTregs at days 5 and 12 of culture demonstrated clustering by cell culture condition and cell type. PC1 reflected the transcriptional differences between nTregs and iTregs; PC2 reflected time in culture (day 5 vs. day 12) (Figure 2D). Notably, transcriptional differences in nTregs as a function of when UHRF1 was deleted were minimal, as clustering remained tight regardless of time in culture and timing of UHRF1 loss. The loss of UHRF1 concurrent with FOXP3 induction (early) in iTregs had a nominal effect (≤3 DEGs) on the iTreg transcriptome when compared with *Uhrf1^+/+^* iTregs. Nevertheless, within PC2, we noted sub-clustering within iTregs on day 12 of culture that reflected the time when UHRF1 was deleted after FOXP3 induction. Pairwise comparison of *Uhrf1^+/+^* and *Uhrf1^fl/fl^* cells from the delayed group analyzed on day 12 revealed 127 DEGs, with genes upregulated in *Uhrf1^+/+^* iTregs including those associated with chemotaxis and migration (*Ccr5*, *Ccr8*, and *S1pr2*) as well as Treg proliferation, differentiation, and transcriptional stabilization (*Skp2*, *Lif*, and *Dusp4*) (Figure 2, E and F, and Supplemental Table 1). Gene set enrichment analysis (GSEA) comparing these iTregs revealed positive enrichment of genes in the *Uhrf1^+/+^* iTregs previously annotated to be upregulated in nTregs and iTregs compared with CD4^+^ Tconv cells in a similar *Foxp3*-GFP IRES construct mouse model as well as an alternate *Foxp3*-GFP chimeric fusion model in which the GFP coding region is inserted in-frame into the N-terminal domain of the *Foxp3* locus, resulting in divergent immunoregulatory functions (Figure 2G) (44–46). This analysis also revealed positive enrichment of genes in *Uhrf1^+/+^* iTregs previously annotated to be upregulated following successful induction of FOXP3 compared with CD4^+^ Tconv cells from the same culture that failed to express FOXP3 (44). *Uhrf1^fl/fl^* iTregs were positively enriched in genes previously annotated to be downregulated in iTregs compared with CD4^+^ Tconv cells (44). Additional GSEA demonstrated positive enrichment of hallmark processes associated with Treg function in *Uhrf1^+/+^* cells, including Myc targets, E2F targets, TGF-β signaling, Wnt/β-catenin signaling, TNF-α signaling via NF-κB, mTORC signaling, KRAS signaling, and IL-2/STAT5 signaling (Supplemental Figure 3D and Supplemental Table 2). No hallmark gene sets were significantly positively enriched in *Uhrf1^fl/fl^* iTregs. To confirm that maintenance DNA methylation was lost upon UHRF1 deletion, we performed genome-wide 5′-cytosine-phosphate-guanine-3′ (CpG) methylation profiling with modified reduced-representation bisulfite sequencing of *Uhrf1^+/+^* and *Uhrf1^fl/fl^* iTregs from both the “early” and “delayed” groups at day 12 of culture. PCA of approximately 80,000 differentially methylated cytosines (FDR *q* < 0.05) revealed distinct clustering according to culture condition (Figure 2H). PC1 reflected methylation changes based on the deletion of UHRF1, whereas PC2 reflected methylation changes based on the timing of UHRF1 deletion. Pairwise comparison of *Uhrf1^+/+^* and *Uhrf1^fl/fl^* iTregs in the delayed group at day 12 demonstrated hypomethylation in *Uhrf1^fl/fl^* iTregs (Figure 2I). Taken together, these results indicate that UHRF1-mediated maintenance DNA methylation is dispensable for the establishment of iTreg FOXP3 expression, transcriptional identity, and suppressive function but is necessary for the subsequent stability of the iTreg transcriptomic signature in vitro.

### UHRF1-deficient iTregs fail to promote recovery following viral pneumonia.

**Figure 3:**
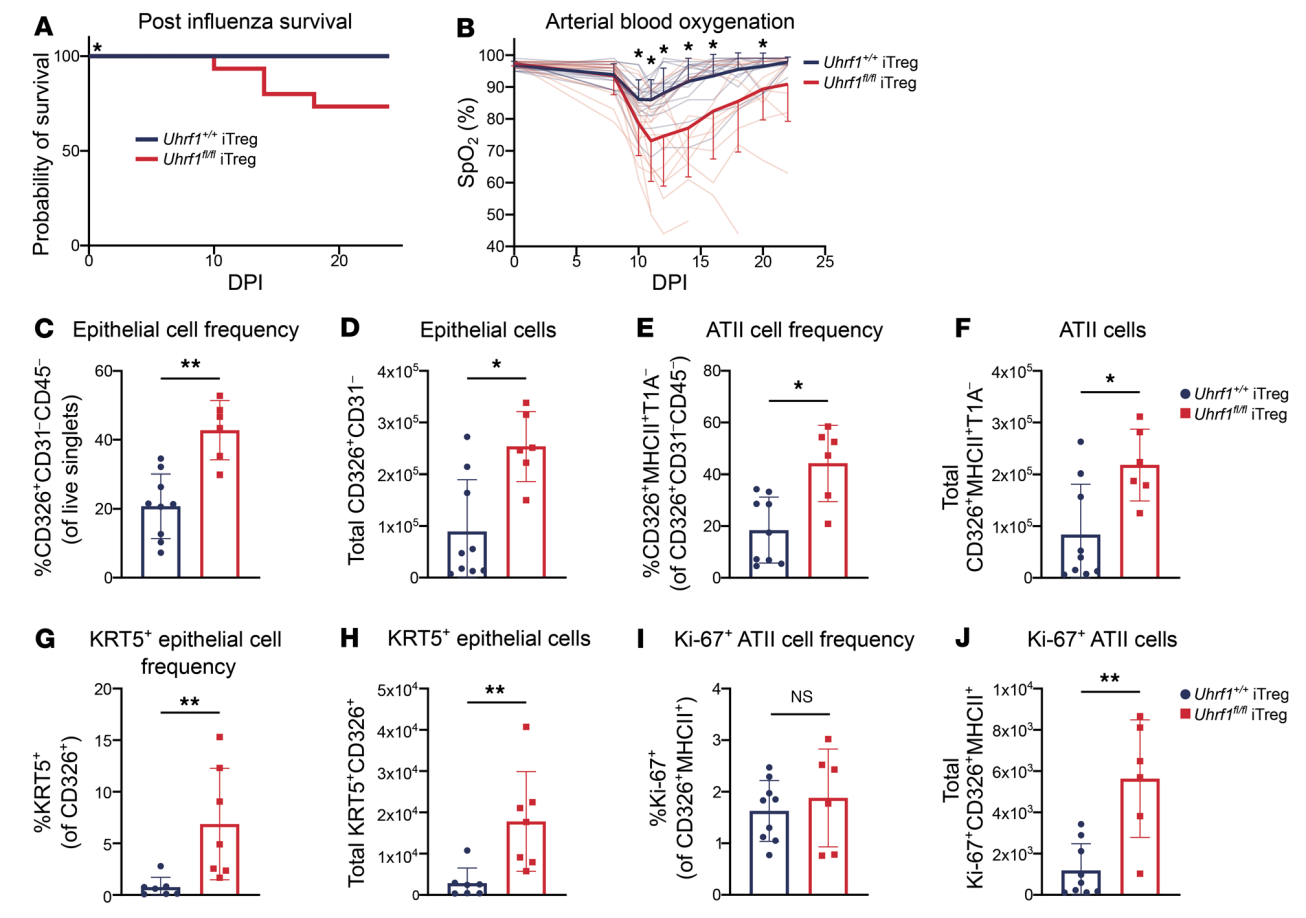
Loss of UHRF1 is sufficient to impair repair capabilities of iTregs during viral pneumonia. *Foxp3^GFP-DTR^* mice were treated with DTx every 48 hours beginning 2 days before inoculation with 6.5 PFU of influenza A/WSN/33 H1N1 virus, and then received retro-orbital adoptive transfer of 1 × 10^6^ *Uhrf1^fl/fl^* or *Uhrf1^+/+^* iTregs at 5 DPI as in Figure 1. iTregs were treated with tamoxifen from culture day 0 to day 3 and harvested for adoptive transfer on culture day 5. Mice were euthanized 24 DPI, and lungs were analyzed by flow cytometry. Epithelial cell data are derived from post-caval lobes. (**A**) Survival of *Foxp3^GFP-DTR^* mice that received *Uhrf1^+/+^* or *Uhrf1^fl/fl^* iTregs. (**B**) SpO_2_ over time in mice from **A**. (**C** and **D**) CD326^+^CD31^–^ cell frequency (**C**) and total number (**D**). (**E** and **F**) CD326^+^MHCII^+^T1A^–^ cell frequency (**E**) and total number (**F**). (**G** and **H**) KRT5^+^CD326^+^ cell frequency (**G**) and total number (**H**). (**I** and **J**) Ki-67^+^CD326^+^MHCII^+^ cell frequency (**I**) and total number (**J**). (**A** and **B**, *Uhrf1^+/+^* iTreg recipients *n* = 18, *Uhrf1^fl/fl^* iTreg recipients *n* = 15; **C**–**F**, *Uhrf1^+/+^* iTreg recipients *n* = 9, *Uhrf1^fl/fl^* iTreg recipients *n* = 6; **G** and **H**, *Uhrf1^+/+^* iTreg recipients *n* = 7, *Uhrf1^fl/fl^* iTreg recipients *n* = 6; **I** and **J**, *Uhrf1^+/+^* iTreg recipients *n* = 9, *Uhrf1^fl/fl^* iTreg recipients *n* = 6.) Survival curve (**A**) *P* value was determined using log-rank (Mantel-Cox) test, *P < 0.05. *P < 0.05 or *q < 0.05 according to mixed-effects model (restricted maximum likelihood) with 2-stage linear step-up procedure of Benjamini, Krieger, and Yekutieli with *Q* = 5% (**B**). Data presented as mean and SD with *P < 0.05, **P < 0.005 according to Mann-Whitney *U* test (**C**–**J**). Data from recipients of *Uhrf1^+/+^* iTregs are re-presented from results shown in Figure 1 and were generated in contemporaneous experiments as the data generated from mice that received *Uhrf1^fl/fl^* iTregs. Data in **A** and **B** generated from 4 independent experiments. Data in **C**–**J** generated from 2 independent experiments.

Because the loss of UHRF1-mediated maintenance DNA methylation promoted transcriptional instability in iTregs, we asked whether the loss of UHRF1 limits the ability of adoptively transferred iTregs to promote recovery following influenza pneumonia. Using the data generated from recipients of UHRF1-sufficient iTregs (*Uhrf1^+/+^*) as a control (presented in Figure 1, B–G) and generated in contemporaneous experiments as the data generated from mice that received UHRF1-deficient (*Uhrf1^fl/fl^*) iTregs, we performed a zoomed-in comparison with recipients of *Uhrf1^fl/fl^* iTregs and found that mice that received *Uhrf1^fl/fl^* iTregs experienced worsened mortality and hypoxemia compared with mice that received *Uhrf1^+/+^* iTregs (Figure 3, A and B). We observed no significant differences in mass recovery (Supplemental Figure 4). Flow cytometry analysis of post-caval lobe single-cell suspensions at 24 DPI revealed a greater frequency and total number of alveolar epithelial cells and alveolar epithelial type II (ATII) cells in *Uhrf1^fl/fl^* iTreg recipients compared with *Uhrf1^+/+^* iTreg recipients (Figure 3, C–F). Notably, compared with recipients of *Uhrf1^+/+^* iTregs, *Uhrf1^fl/fl^* iTreg recipients also displayed a greater frequency and total number of KRT5^+^ epithelial cells and a higher total number, but not frequency, of Ki-67^+^ ATII cells, suggesting a greater degree of peak injury in the recipients of *Uhrf1^fl/fl^* iTregs (Figure 3, G–J). Collectively, adoptive transfer of UHRF1-deficient iTregs compromised recovery from viral pneumonia, and recipients of *Uhrf1^fl/fl^* iTregs displayed evidence of a greater degree of peak injury.

### Adoptive transfer of UHRF1-deficient iTregs results in delayed repair of lung injury following viral pneumonia compared with UHRF1-sufficient iTregs.

**Figure 4:**
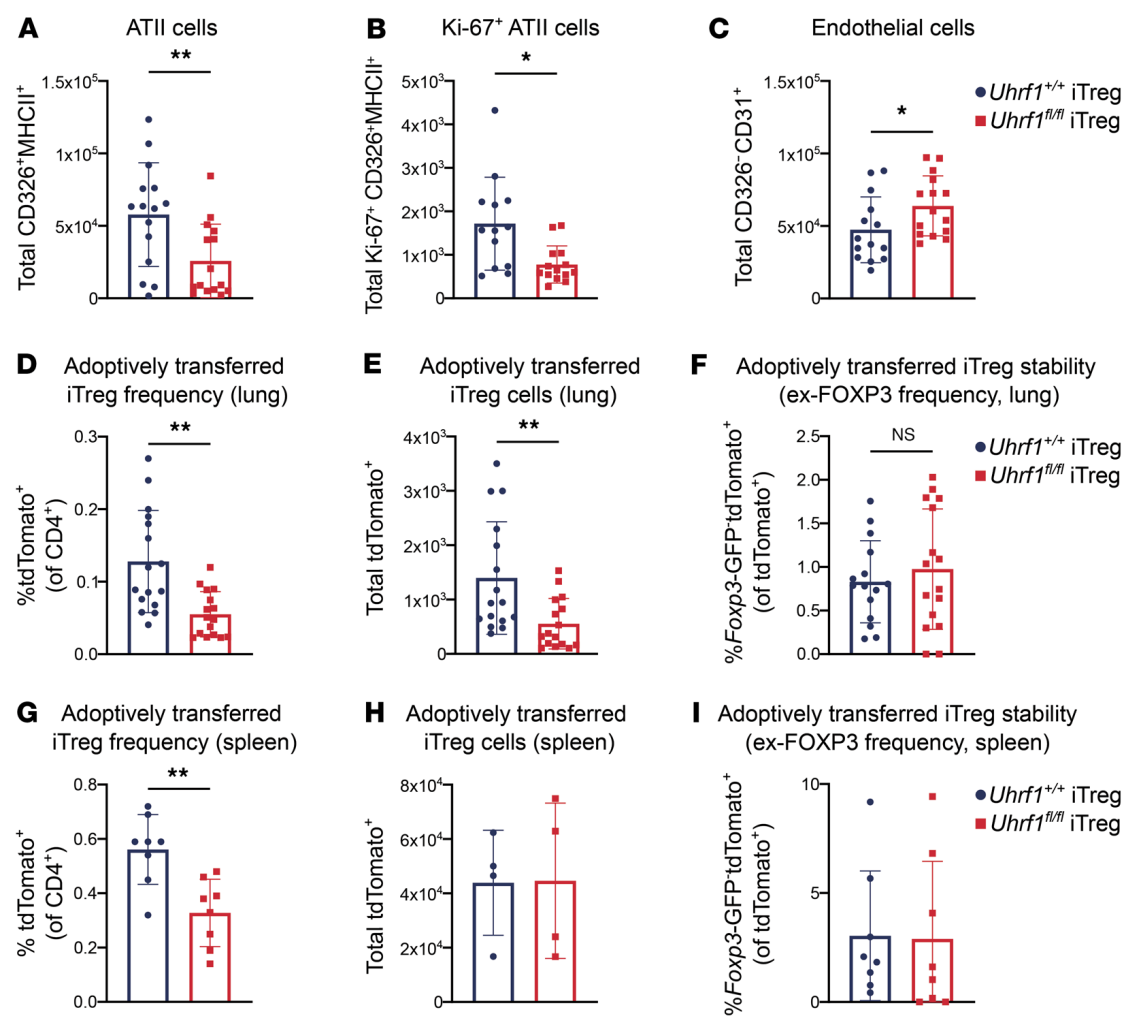
UHRF1-deficient iTregs promote an insufficient tissue-protective response during peak lung injury. *Foxp3^GFP-DTR^* mice were treated with DTx every 48 hours beginning 2 days before inoculation with 6.5 PFU of influenza A/WSN/33 H1N1 virus and received retro-orbital adoptive transfer of 1 × 10^6^ *Uhrf1^fl/fl^* or *Uhrf1^+/+^* iTregs at 5 DPI. iTregs were treated with tamoxifen from day 0 to day 3 of culture and harvested for adoptive transfer on culture day 5. Recipient mice were euthanized 11 DPI, and lungs and spleen were analyzed by flow cytometry. Epithelial cell data are derived from the post-caval lobes. Transferred iTreg quantification is derived from the remaining lung lobes. (**A**) Total number of ATII cells (*n* = 15 per group). (**B**) Total number of Ki-67^+^ ATII cells (*Uhrf1^+/+^* iTregs *n* = 13, *Uhrf1^fl/fl^* iTregs *n* = 14). (**C**) Total number of CD326^–^CD31^+^ (endothelial) cells (*Uhrf1^+/+^* iTregs *n* = 14, *Uhrf1^fl/fl^* iTregs *n* = 15). (**D**) Frequency of tdTomato^+^ cells in lung (*n* = 16 per group). (**E**) Total number of tdTomato^+^ cells in lung (*n* = 16 per group). (**F**) Frequency of *Foxp3*-GFP^–^tdTomato^+^ (ex-FOXP3) cells in lung (*Uhrf1^+/+^* *n* = 15, *Uhrf1^fl/fl^* *n* = 16). (**G**) Frequency of tdTomato^+^ cells in spleen (*n* = 8 per group). (**H**) Total number of tdTomato^+^ cells in spleen (*n* = 4 per group). (**I**) Frequency of *Foxp3*-GFP^–^tdTomato^+^ cells in spleen (*n* = 8 per group). Data presented as mean and SD. *P < 0.05, **P < 0.005, according to Mann-Whitney *U* test. Data in **A**–**F** generated from 2 independent experiments. Data in **G**–**I** generated from 1 independent experiment.

In addition to promoting repair, Tregs possess distinct tissue-protective properties that impart resilience to damage (47). As analysis of our adoptive transfer experiments at a late repair time point (24 DPI) demonstrated a reduced frequency of ATII cells in recipients of *Uhrf1^+/+^* iTregs compared with recipients of *Uhrf1^fl/fl^* iTregs despite reduced hypoxemia and mortality, we hypothesized that *Uhrf1^+/+^* iTregs impart resilience to lung injury by decreasing infiltration by inflammatory immune cells or by dampening early injury to result in a less robust repair response later in the disease course. We therefore performed an additional series of adoptive transfer experiments with analysis at 11 DPI, a time point that correlated with peak lung injury. Like in experiments analyzed at 24 DPI, *Uhrf1^+/+^* and *Uhrf1^fl/fl^* iTregs were treated with tamoxifen for 3 days, and cells were harvested for adoptive transfer on culture day 5. We found no difference in immune cell infiltration, including total leukocytes and myeloid and lymphoid cell subsets (Supplemental Figure 5, A–F). In contrast, lungs from mice that received *Uhrf1^+/+^* iTregs displayed a significantly greater total number of ATII cells with a concomitantly greater total number of Ki-67^+^ ATII cells compared with lungs from recipients of *Uhrf1^fl/fl^* iTregs (Figure 4, A and B). No significant differences were observed in total numbers of epithelial or KRT5^+^ epithelial cells (Supplemental Figure 5, G and H). Interestingly, recipients of *Uhrf1^fl/fl^* iTregs displayed a significantly greater total number of endothelial cells when directly compared with recipients of *Uhrf1^+/+^* iTregs (Figure 4C). We quantified the adoptively transferred tdTomato^+^ iTregs and found a significantly lower frequency and total number of *Uhrf1^fl/fl^* iTregs in the lungs compared with *Uhrf1^+/+^* iTregs (Figure 4, D and E). No significant difference was observed in the frequency of ex-FOXP3 cells between groups (Figure 4F). To assess whether the inflammatory microenvironment of the lung could be influencing the differences in engraftment following adoptive transfer, we quantified tdTomato^+^ iTregs from the spleens of these mice and found that the frequency but not the total number of iTregs was lower in recipients of *Uhrf1^fl/fl^* compared with *Uhrf1^+/+^* iTregs (Figure 4, G and H). Consistent with data from the lungs, no difference was observed in the frequency of ex-FOXP3 cells in the spleen (Figure 4I). Taken together with the data from 24 DPI, these results suggest that UHRF1-deficient iTregs provide an insufficient early tissue-protective response that results in delayed repair.

To differentiate between an intrinsic difference in functionality of *Uhrf1^fl/fl^* iTregs and dysfunctionality due to their diminished engraftment, we performed an additional adoptive transfer experiment in which we attempted to equalize the number of engrafted cells. Based on differences we observed in the total number and frequency of cells between *Uhrf1^+/+^* and *Uhrf1^fl/fl^* iTregs in the lungs at 11 DPI, we estimated that adoptive transfer of 4 × 10^6^ *Uhrf1^fl/fl^* iTregs would be sufficient. Mice that received 1 × 10^6^ *Uhrf1^+/+^* iTregs or 1 × 10^6^ *Uhrf1^fl/fl^* iTregs served as positive and negative controls, respectively. Intriguingly, and consistent with prior experiments, mice that received 1 × 10^6^ *Uhrf1^+/+^* iTregs exhibited significantly greater SpO_2_ compared with mice that received either quantity of *Uhrf1^fl/fl^* iTregs (Supplemental Figure 6A). Recipients of 1 × 10^6^ *Uhrf1^+/+^* iTregs exhibited a trend toward greater survival (Supplemental Figure 6B). Mice that received 1 × 10^6^ *Uhrf1^+/+^* iTregs also exhibited reduced mass loss over the course of influenza (Supplemental Figure 6C). Despite the 4-fold difference in adoptively transferred *Uhrf1^fl/fl^* iTregs, quantification of adoptively transferred iTregs in the lungs and spleens of influenza-infected mice at 24 DPI revealed a significantly lower frequency and total number of *Uhrf1^fl/fl^* iTregs and, unlike at 11 DPI, a concomitantly larger *Foxp3-*GFP^–^tdTomato^+^ ex-FOXP3 population in recipients of *Uhrf1^fl/fl^* iTregs (Supplemental Figure 6, D–I). Nevertheless, the quantity of *Uhrf1^fl/fl^* iTregs in recipients of 4 × 10^6^ *Uhrf1^fl/fl^* iTregs was significantly higher than that in recipients of 1 × 10^6^ *Uhrf1^fl/fl^* iTregs. Taken together, these results suggest that the inability to equalize the number of engrafted iTregs in a linear fashion by administering a higher adoptive transfer dose further supports a substantial defect in recruitment attributable to the loss of UHRF1.

### UHRF1-deficient iTregs display transcriptional instability and poor engraftment after adoptive transfer into mice with viral pneumonia.

**Figure 5:**
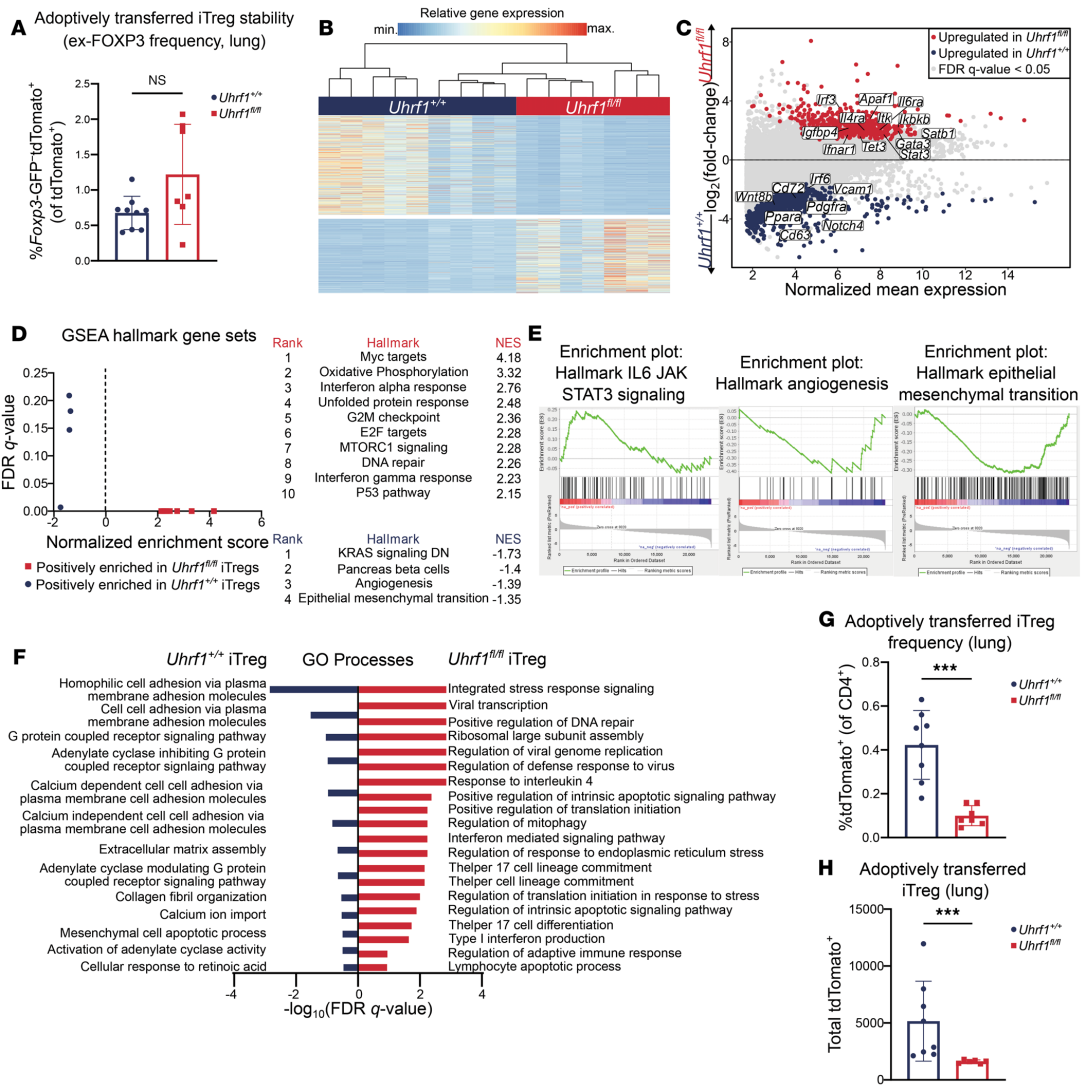
UHRF1 is required for iTreg phenotypic stability and lung tissue engraftment following viral pneumonia. DTx-treated, influenza A–infected *Foxp3^GFP-DTR^* recipient mice received retro-orbital adoptive transfer of 1 × 10^6^ *Foxp3*-GFP^+^tdTomato^+^ *Uhrf1^fl/fl^* or *Uhrf1^+/+^* iTregs at 5 DPI. iTregs were cultured with tamoxifen from day 0 to day 3, then harvested for adoptive transfer, as in Figure 1. Transferred *Foxp3*-GFP^+^tdTomato^+^ *Uhrf1^fl/fl^* or *Uhrf1^+/+^* iTregs were sorted from the lungs of recipient *Foxp3^GFP-DTR^* mice 24 DPI for quantification and profiling via bulk RNA-Seq. (**A**) Frequency of *Foxp3*-GFP^–^tdTomato^+^ (ex-FOXP3) cells. (**B**) *K*-means clustering of 1,187 genes with FDR *q* < 0.05 comparing recovered *Uhrf1^+/+^* and *Uhrf1^fl/fl^* iTregs with *k* = 2. (**C**) MA plot comparing gene expression of recovered *Uhrf1^+/+^* and *Uhrf1^fl/fl^* iTregs. Genes of interest are annotated. (**D**) GSEA dot plot highlighting key statistics (FDR *q* value and normalized enrichment score [NES]) and enriched gene sets. Red dots denote gene sets with a positive enrichment score or enrichment at the top of the ranked list. Blue dots denote gene sets with a negative enrichment score or enrichment at the bottom of the ranked list. (**E**) Enrichment plots of hallmark gene sets generated through GSEA pre-ranked testing of the expressed genes in *Uhrf1^+/+^* and *Uhrf1^fl/fl^* iTregs. All gene sets displayed significant enrichment with FDR *q* value < 0.25. (**F**) Selected Gene Ontology (GO) processes from 945 and 105 total enriched gene sets with FDR *q* < 0.25 in *Uhrf1^fl/fl^* and *Uhrf1^+/+^* iTregs, respectively. Gene sets are annotated and ranked by –log_10_-transformed FDR *q* value. (**G**) Frequency of tdTomato^+^ iTregs recovered. (**H**) Total number of tdTomato^+^ iTregs recovered. ***P < 0.0005 according to Mann-Whitney *U* test (**A**–**F**, *Uhrf1^+/+^* *n* = 9, *Uhrf1^fl/fl^* *n* = 7; **G**, *Uhrf1^+/+^* *n* = 8, *Uhrf1^fl/fl^* *n* = 7; **H**, *Uhrf1^+/+^* *n* = 8, *Uhrf1^fl/fl^* *n* = 6). Data generated from 2 independent experiments.

To explore cell-intrinsic mechanisms underlying the loss of pro-recovery function in UHRF1-deficient iTregs, we profiled *Foxp3*-GFP^+^tdTomato^+^ iTregs sorted from the lungs of *Uhrf1^+/+^* and *Uhrf1^fl/fl^* iTreg recipients at 24 DPI. The frequency of adoptively transferred cells that had lost FOXP3 expression (*Foxp3*-GFP^–^TdTomato^+^; ex-FOXP3 cells) was not significantly different between *Uhrf1^+/+^* and *Uhrf1^fl/fl^* iTregs, though the trend reflects a larger ex-FOXP3 population in *Uhrf1^fl/fl^* iTregs as seen in the data presented in Supplemental Figure 6 (Figure 5A). Looking beyond stability at the FOXP3 locus, we subsequently focused our analysis on transcriptomic comparison of *Uhrf1^+/+^* and *Uhrf1^fl/fl^* iTregs. We identified 1,187 DEGs; *k*-means clustering of these DEGs identified 2 distinct clusters (Figure 5B and Supplemental Table 1). Genes upregulated in *Uhrf1^+/+^* iTregs included several associated with Treg pro-repair function or tissue repair, such as *Notch4*, *Dll4*, *Pdgfra*, *Mmp12*, *Fgfr1*, *Loxl2*, *Wnt8b*, and *Yap1* (Figure 5C). In contrast, several genes upregulated in *Uhrf1^fl/fl^* iTregs are associated with effector helper T cell lineage commitment, such as *Stat3*, *Il6ra*, *Jak1*, *Itk*, *Igfbp4*, *Zeb1*, *Gata3*, *Il4ra*, and *Ifnar1*. GSEA revealed positive enrichment of gene sets associated with IL-6/STAT3 signaling as well as negative enrichment of genes associated with angiogenesis and epithelial-mesenchymal transition in *Uhrf1^fl/fl^* iTregs (Figure 5, D and E, and Supplemental Table 3). Functional enrichment analysis revealed upregulation of gene sets associated with protein translation, interferon and interleukin signaling, helper T cell and Th17 differentiation, viral processes, DNA damage repair, cellular stress responses, and apoptotic processes in *Uhrf1^fl/fl^* iTregs (Figure 5F and Supplemental Table 3). We quantified the number of transferred iTregs in the lungs of recipient mice at 24 DPI and again found a significantly reduced frequency and total number of *Uhrf1^fl/fl^* iTregs compared with *Uhrf1^+/+^* iTregs (Figure 5, G and H).

To assess for extrapulmonary signatures due to the loss of UHRF1, we sorted *Foxp3*-GFP^+^tdTomato^+^ and *Foxp3*-GFP^–^tdTomato^+^ (ex-FOXP3) cells from the spleens of *Uhrf1^+/+^* and *Uhrf1^fl/fl^* iTreg recipients at 24 DPI for gene expression profiling. PCA of 457 DEGs identified following ANOVA-like testing with FDR *q* < 0.05 demonstrated clustering by genotype and FOXP3 expression (Supplemental Figure 7A). PC1 reflected the transcriptional differences dependent on FOXP3 expression, and PC2 reflected differences between genotype (*Uhrf1^+/+^* vs. *Uhrf1^fl/fl^*). Pairwise comparison of *Uhrf1^+/+^* and *Uhrf1^fl/fl^* FOXP3^+^ cells revealed 183 DEGs, with genes upregulated in *Uhrf1^+/+^* iTregs associated with cell cycle regulation/cellular proliferation and induction and maintenance of Treg function (*E2f3*, *Ncoa3*, *Hpse*) (Supplemental Figure 7, B and C, and Supplemental Table 1). Genes upregulated in *Uhrf1^fl/fl^* iTregs included those associated with maintenance of function but also proinflammatory cytokines and cytokine receptors (*Mst1*, *Tmed4*, *Il1b*, *Il17rb*, and *Il4*). Pairwise comparison of *Uhrf1^+/+^* and *Uhrf1^fl/fl^* ex-FOXP3 cells revealed 274 DEGs, with genes upregulated in *Uhrf1^+/+^* ex-FOPX3 cells associated with Treg stability and suppressive function (*Ikzf2*, *Zap70*, *Tnfrsf9*, *Il1r1*, and *Parp11*). Genes upregulated in *Uhrf1^fl/fl^* ex-FOXP3 cells included some associated with alternate effector T cell function and apoptosis (*Il17rb*, *Il13*, *Crtc2*, *Casp8ap2*, and *Tnfrsf8*) (Supplemental Figure 7, D and E, and Supplemental Table 1).

To further elucidate whether engraftment was influenced by the inflammatory microenvironment of the lung, we harvested adoptively transferred *Foxp3*-GFP^+^tdTomato^+^ cells from the spleens and lungs of *Foxp3^GFP-DTR^* recipients of *Uhrf1^+/+^* or *Uhrf1^fl/fl^* iTregs that received DTx but not influenza. We found that iTregs in recipients of *Uhrf1^+/+^* iTregs were consistently greater in frequency and total number than those in recipients of *Uhrf1^fl/fl^* iTregs and, intriguingly, displayed a significantly greater degree of instability (ex-FOXP3 cells) in the lungs but not the spleen (Supplemental Figure 8, A–G). Collectively, these data suggest that iTregs require UHRF1 to stabilize their phenotypic identity, upregulate repair processes, and promote tissue engraftment following influenza virus pneumonia.

### Loss of UHRF1-mediated maintenance DNA methylation results in disrupted DNA methylation and delayed expression of signature Treg transcriptional programs.

**Figure 6:**
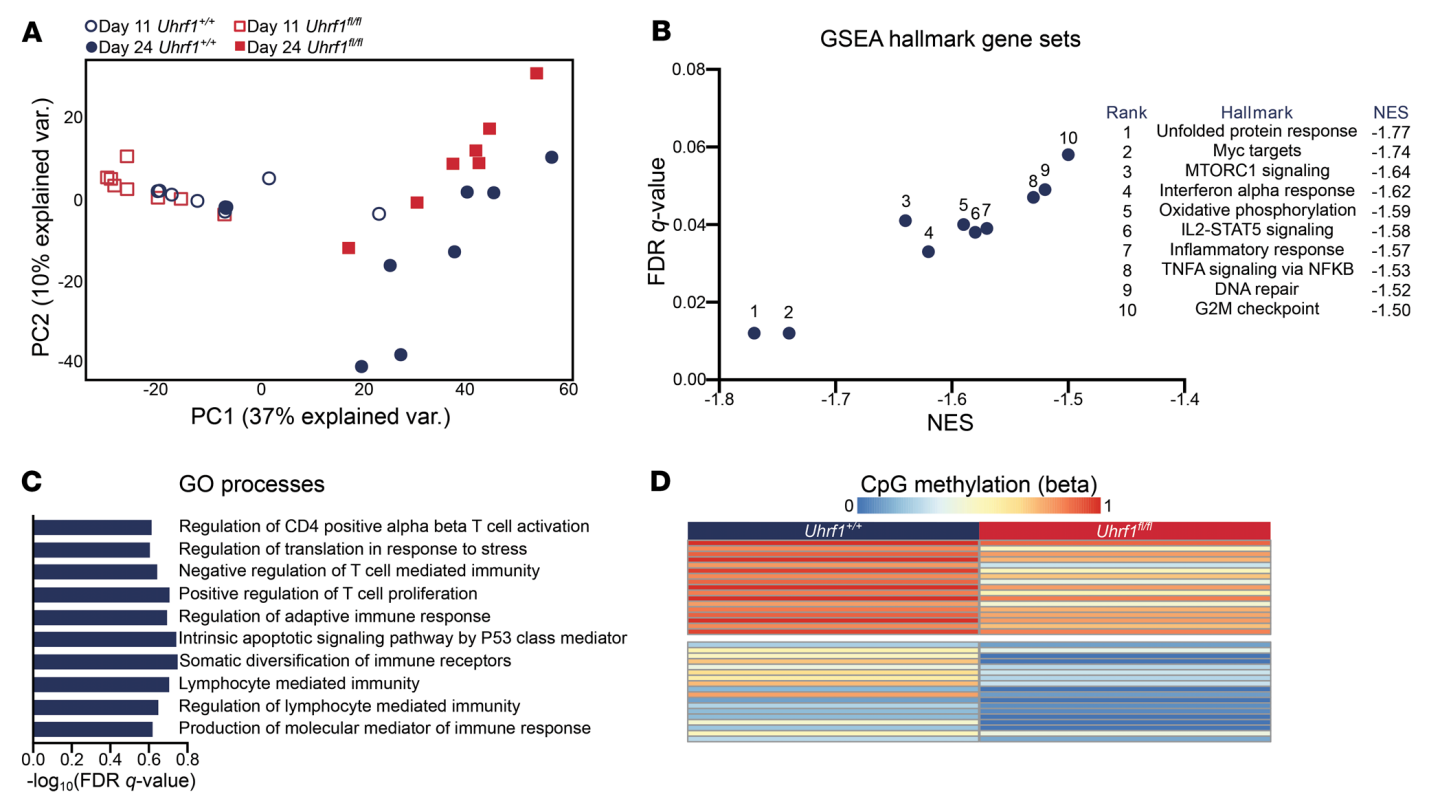
Loss of UHRF1 results in delayed transcriptional changes over the course of viral pneumonia. *Foxp3^GFP-DTR^* mice were treated with DTx every 48 hours beginning 2 days before inoculation with 6.5 PFU of influenza A/WSN/33 H1N1 virus and then received retro-orbital adoptive transfer of 1 × 10^6^ *Foxp3*-GFP^+^tdTomato^+^ *Uhrf1^fl/fl^* or *Uhrf1^+/+^* iTregs at 5 DPI as in Figure 1. iTregs were treated with tamoxifen from day 0 to day 3 of culture, then harvested for adoptive transfer on culture day 5. Adoptively transferred *Foxp3*-GFP^+^tdTomato^+^ *Uhrf1^fl/fl^* or *Uhrf1^+/+^* iTregs were sorted from the lungs of recipient *Foxp3^GFP-DTR^* mice 11 or 24 DPI and were compared via RNA transcriptomic and DNA methylation analysis. (**A**) PCA of 2,117 DEGs identified from ANOVA-like testing with FDR *q* < 0.05. Independent biological replicates are shown. (**B**) GSEA dot plot highlighting key statistics (FDR *q* value and NES) and enriched gene sets for *Uhrf1^+/+^* iTregs at 11 DPI. Blue dots denote gene sets with a negative enrichment score or enrichment at the bottom of the ranked list. (**C**) Selection of representative enriched GO processes from 40 total enriched gene sets with an FDR *q* < 0.25 in *Uhrf1^+/+^* iTregs at 11 DPI. Gene sets are annotated and ranked by –log_10_-transformed FDR *q* value. (**D**) *K*-means clustering of 34 differentially methylated regions with a difference of ≥10% between *Uhrf1^+/+^* and *Uhrf1^fl/fl^* iTregs. (**A**–**D**, 24 DPI *Uhrf1^+/+^* iTreg recipients *n* = 9, *Uhrf1^fl/fl^* iTreg recipients *n* = 7, 11 DPI *Uhrf1^+/+^* and *Uhrf1^fl/fl^* iTreg recipients *n* = 8).

To determine transcriptional differences in iTregs earlier in the course of injury and assess for differences in the transcriptional landscape over time, we compared transcriptional profiling of sorted *Foxp3*-GFP^+^tdTomato^+^ cells from the lungs at 11 and 24 DPI from DTx-treated, influenza-infected mice that received adoptive transfer of *Uhrf1^fl/fl^* or *Uhrf1^+/+^* iTregs at 5 DPI. PCA of 2,117 DEGs identified following ANOVA-like testing with FDR *q* < 0.05 demonstrated clustering by DPI and genotype (Figure 6A). PC1 reflected the transcriptional differences between iTregs at 11 versus 24 DPI, and PC2 reflected differences between genotypes (*Uhrf1^+/+^* vs. *Uhrf1^fl/fl^*) at 24 DPI. Pairwise comparison of *Foxp3*-GFP^+^tdTomato^+^ cells isolated at 11 DPI revealed 32 DEGs. GSEA revealed positive enrichment of hallmark processes associated with essential Treg functions, including Myc targets, oxidative phosphorylation, E2F targets, TNF-α signaling via NF-κB, mTORC signaling, and IL-2/STAT5 signaling in *Uhrf1^+/+^* iTregs. No processes were positively enriched in *Uhrf1^fl/fl^* iTregs at 11 DPI (Figure 6B and Supplemental Table 4). Additional GSEA revealed a similar pattern, with positive enrichment of Gene Ontology processes seen in *Uhrf1^+/+^* iTregs including protein translation, T cell differentiation, regulation of lymphocyte-mediated immunity, DNA damage repair, cellular stress responses, and apoptotic processes, but no positively enriched processes in *Uhrf1^fl/fl^* iTregs (Figure 6C and Supplemental Table 4). An unsupervised analysis comparing differentially methylated regions of *Uhrf1^fl/fl^* and *Uhrf1^+/+^* iTregs at 24 DPI with at least a 10% difference in methylation demonstrated disrupted methylation at 34 regions in *Uhrf1^fl/fl^* iTregs (Figure 6D and Supplemental Table 1). These findings fit a pattern in which processes enriched in *Uhrf1^+/+^* iTregs at 11 DPI are not enriched in *Uhrf1^fl/fl^* iTregs until 24 DPI, suggesting a delayed transcriptomic phenotype paralleling the delayed repair phenotype of the recipient mice.

## Discussion

Natural Tregs (nTregs) depend on specific patterns of DNA methylation to establish cell identity and stability as well as to exert their pro-recovery function following acute lung injury (8, 12, 30, 48). Here, we demonstrated the ability of transferred iTregs to promote recovery following viral pneumonia in mice. We further demonstrated that maintenance DNA methylation mediated by the epigenetic regulator UHRF1 is necessary for iTreg reparative function during viral pneumonia. Recipients of UHRF1-deficient iTregs experienced worsened hypoxemia and mortality with dysregulated and delayed lung repair. In vitro, the loss of maintenance DNA methylation resulted in downregulation of chemokine receptor gene expression, such as *Ccr4*, *Ccr5*, and *Ccr8*. In vivo, UHRF1-deficient iTregs displayed reduced lung tissue engraftment, greater propensity for instability in FOXP3 expression, and downregulation of genes associated with pro-repair function and upregulation of pro-apoptotic genes and of transcription factors defining alternate effector T cell lineage. These findings support the use of iTregs for cellular therapy while demonstrating the necessity of maintenance DNA methylation in iTreg stability and function.

Our observations support a paradigm in which critical changes occur across the DNA methylation landscape between the CD4^+^ stage of T cell development and subsequent FOXP3 expression that cause a differential effect of the loss of maintenance DNA methylation on iTreg function. We demonstrated that iTregs that lose UHRF1 following FOXP3 induction possess a reduced ability to protect the lung from alveolar epithelial injury, leading to delayed lung repair. In contrast, other studies demonstrate that iTregs generated from UHRF1-deficient CD4^+^ T cells exhibit hypersuppressive function when adoptively transferred into lymphocyte-deficient mice with Tconv cell–mediated colitis (31). In both cases, the loss of UHRF1 did not affect FOXP3 induction, yet here, the loss of UHRF1 resulted in greater instability in vivo while also altering the transcriptome at other loci. A similar effect dependent on the timing of loss of UHRF1 exists in nTregs (30, 49). Indeed, work from our group demonstrated that the loss of UHRF1 at the FOXP3^+^ stage of nTreg development caused the spontaneous onset of widespread scurfy-like inflammation (30). Intriguingly, in that study, we also observed the generation of ex-FOXP3 cells following the induced loss of UHRF1 in vivo, suggesting a link between UHRF1-mediated maintenance DNA methylation and the stability of FOXP3 expression in Tregs. In contrast, mice with pan–T cell UHRF1 deficiency develop inflammation localized specifically to the colon (49). Although the latter study noted differences in thymic and peripheral Treg populations, it further serves as evidence of the differential effect of the loss of UHRF1 depending on the developmental stage of the Treg.

Despite reports of instability of iTregs within inflammatory microenvironments, our data suggest that iTregs retain their function in promoting lung repair, like adoptively transferred nTregs (12, 16–18). Suppressive mechanisms did not appear to play a role in the differential effects of the loss of UHRF1, as immune infiltration and activation were similar in mice that received UHRF1-sufficient and -deficient iTregs. Whether and how the inflammatory microenvironment contributed to reduced engraftment of UHRF1-deficient iTregs is unclear. RNA-Seq data from cells cultured in vitro suggest that reduced engraftment may be a result of lower expression of homing receptors, such as CCR4, CCR5, and CCR8. In addition, data from UHRF1-deficient iTregs recovered from the lung revealed upregulation of several pro-apoptotic factors, suggesting an influence from the inflammatory microenvironment that reduced UHRF1-deficient iTreg numbers.

iTregs may therefore serve as a practical, safe, and efficient alternative to nTreg cellular therapy for severe, rapidly progressive, tissue-injurious inflammatory diseases, such as acute respiratory distress syndrome (50). Currently, nTregs are obtained via leukapheresis from autologous peripheral or allogeneic cord blood, and protocols to expand them are on the order of weeks (51, 52). This same process could be applied to iTregs in a fraction of the time, as they derive from CD4^+^ Tconv cells and expand rapidly in culture, allowing for intervention at an earlier time point in the disease course. Similar to strategies for nTregs, ex vivo modification strategies could be implemented to further enhance iTreg therapeutic efficacy (15). As we noted that cellular engraftment may play a role in the differential effects observed in recipients of UHRF1-sufficient and -deficient iTregs, in vitro supplementation with factors to promote homing to the site of injury via enhanced chemokine receptor expression could ensure arrival of the transferred cell product to the site of injury. Additionally, because the loss of UHRF1-mediated maintenance DNA methylation resulted in upregulation of genes associated with effector helper T cell lineage commitment, factors to promote their downregulation could be leveraged to ensure stable functioning.

In summary, our data establish that iTregs promote timely repair of damaged lung tissue following viral pneumonia. Mechanistically, UHRF1-mediated maintenance DNA methylation is required for optimal iTreg engraftment and reparative function. These data credential iTregs as potential cellular therapy to promote repair following viral pneumonia–induced acute lung injury.

## Methods

### Sex as a biological variable.

In preliminary experiments using the influenza model in wild-type mice, we found that female mice experienced higher mortality than males. As recipients of *Uhrf1^fl/fl^* iTregs experienced higher mortality than controls, male mice were used for these experiments. Hence, sex was not considered as a biological variable in all experiments.

### Mice.

All mice were housed and used in accordance with the Institutional Animal Care and Use Committee (IACUC) at Northwestern University. Animals received water ad libitum, were housed at a temperature range of 20°C–23°C under 14-hour light/10-hour dark cycles, and received standard rodent chow. For all experiments, male mice between 8 and 20 weeks of age were used. *Foxp3^GFP-DTR^* mice were purchased from The Jackson Laboratory (strain 016958) and bred on-site. *Foxp3^Cre^* mice were purchased from The Jackson Laboratory (strain 016959) and bred on-site. C57BL/6 *Uhrf1^fl/fl^Foxp3^GFP-CreERT2^Rosa26Sor^CAG-tdTomato^* or *Foxp3^GFP-CreERT2^Rosa26Sor^CAG-tdTomato^* mice were generated as previously described (30). All animals were genotyped using services provided by Transnetyx Inc., with primer sequences provided by The Jackson Laboratory or published in prior work (30).

### Diphtheria toxin, influenza A virus administration, and adoptive transfer.

To ablate the endogenous mouse Treg population, lyophilized diphtheria toxin (List Biological Laboratories, product 150) was resuspended in sterile PBS and administered in 100 μL via intraperitoneal injection every 2 days to *Foxp3^GFP-DTR^* mice. The initial loading dose was 50 μg/kg followed by maintenance dosing of 10 μg/kg. For influenza A virus administration, mice were intubated while under isoflurane anesthesia using a 20-gauge angiocatheter cut to a length that placed the tip of the catheter above the carina. After intubation, a 1 mL syringe containing 50 μL of sterile PBS and with the plunger removed was attached onto the angiocatheter to act as a spirometer and confirm proper placement into the trachea. Once proper placement was confirmed, the mice were then instilled with mouse-adapted influenza A/WSN/33 H1N1 virus in 50 μL of sterile PBS as previously described (33, 40, 53–55). A 300 μL bolus of air from a second 1 mL syringe was ejected into the angiocatheter to clear it and ensure the bolus of influenza was well distributed into the lungs. nTregs were adoptively transferred directly after isolation from the spleen and lymph nodes of 8- to 12-week-old *Foxp3^GFP-CreERT2^Rosa26Sor^CAG-tdTomato^* mice using the mouse Miltenyi Biotec CD4^+^CD25^+^ Regulatory T Cell Isolation Kit (catalog 130-091-041). For Tconv cell and iTreg adoptive transfer, cells were harvested from culture on day 5, restained for CD4 and viability dye, and sorted via a microfluidics MACSQuant Tyto sorter (Miltenyi Biotec) to enhance purity (Supplemental Table 5). Cells were subsequently washed twice and resuspended in sterile PBS at a concentration of 1 × 10^6^ cells/100 μL. Once resuspended, cells were drawn up into a 500 μL insulin syringe (Becton Dickinson 329461) and delivered into mice placed under isoflurane anesthesia via the retro-orbital sinus (40).

### Cell culture and preparation for adoptive transfers.

To prepare Tconv cells and iTregs for cell culture prior to adoptive transfer, spleens and lymph nodes (inguinal and axillary) were harvested from adult (8- to 12-week-old) mice, disrupted using scored 60 mm Petri dishes in PBS, and filtered through a 40 μm nylon mesh filter to obtain single-cell suspensions. Red blood cell (RBC) lysis was performed using Gibco ACK lysing buffer (catalog A1049201). Cell counts were obtained with a Cellometer K2 Counter using AOPI stain (Nexcelom Bioscience, catalog SD014-0106). CD4^+^ T cells were purified from single-cell suspension using the EasySep mouse CD4^+^ T cell Isolation Kit (Stemcell, catalog 19852) or the negative fraction of the Miltenyi Biotec mouse CD4^+^CD25^+^ Regulatory T cell Isolation Kit (catalog 130-091-041) according to the manufacturer’s instructions. To further enhance purity before culture, enriched CD4^+^ single-cell suspensions were stained with antibodies against CD4 for flow cytometry sorting (Supplemental Table 5). CD4^+^ Tconv cells were separated from CD4^+^*Foxp3*-GFP^+^ nTregs with a microfluidics MACSQuant Tyto sorter (Miltenyi Biotec). Dead cells were excluded using a viability dye for analysis and sorting (Supplemental Table 5) in all experiments. For Tconv cell and iTreg cell culture, 300,000 sorted CD4^+^*Foxp3*-GFP^–^ Tconv cells were seeded in 24-well plates (Fisher Scientific, FB012929) coated with αCD3ε/αCD28 antibody (BD Pharmingen catalog 553058 and 553295) at concentrations of 3 μg/mL and 5 μg/mL, respectively. Recombinant human IL-2 was added to the Tconv cell culture at 50 U/mL. To generate iTregs, recombinant human IL-2 and TGF-β (PeproTech, catalog 100-21-10UG) were added at 50 U/mL and 10 ng/mL, respectively. All cell types were cultured in RPMI 1640 (Gibco, catalog 11875-093) medium supplemented with 10% fetal bovine serum, 5 mM HEPES (Gibco, catalog 15630080), 100 U/mL penicillin-streptomycin (Gibco, catalog 15140122), 1 mM Na-pyruvate (Gibco, catalog 11360-070), 100 mM MEM non-essential amino acids (Gibco, catalog 11140050), 2 mM l-glutamine (Gibco, catalog 25030081), and 50 μM 2-mercaptoethanol (Sigma-Aldrich, catalog M3148). For FOXP3^+^ cell lineage tracing and *Foxp3*-Cre–mediated loss of UHRF1, (Z)-4-hydroxytamoxifen (Sigma-Aldrich, catalog H7904) was added to cell culture from day 0 to day 3 at a concentration of 500 nM. Tconv cells and iTregs were removed from antibody-coated plates on day 3 of culture and passaged into fresh, non-coated plates, and additional recombinant human IL-2 and TGF-β were added at 50 U/mL and 10 ng/mL, respectively.

### Measurement of physiologic readouts of viral pneumonia progression and recovery.

To prepare mice for arterial oxyhemoglobin saturation (SpO_2_) measurement, 1 day before influenza inoculation, a depilatory cream containing the active ingredients potassium thioglycolate (4%) and calcium hydroxide (1.5%) (Nair) was applied to the hair on the dorsal neck for 2 minutes and subsequently gently removed with wet gauze under isoflurane anesthesia. A MouseOx Plus pulse oximeter (Starr Life Sciences) was used to measure SpO_2_. Unanesthetized mice were immobilized, and an oximeter collar clip was secured to the hairless neck. Baseline SpO_2_ and mass measurements were obtained before influenza administration with subsequent measurements taken every other day after inoculation.

### Lung tissue harvesting, processing, and analysis.

Mice were euthanized using a carbon dioxide euthanasia chamber followed by cervical dislocation and slowly infused with HBSS through the right atrium of the heart to clear the pulmonary circulation of blood. The lungs were then harvested, and the larger airways and other mediastinal structures were trimmed. The post-caval lobes were removed and set aside for epithelial cell analysis by flow cytometry. For adoptively transferred iTreg and infiltrating immune cell analysis, the remaining lung lobes were grossly homogenized with scissors in HBSS containing 2 mg of collagenase D (Sigma-Aldrich, catalog 11088866001) and 0.25 mg of DNase I (Sigma-Aldrich, catalog 10104159001) per milliliter. The lung suspensions were subsequently incubated for 45 minutes at 37°C and then further homogenized with a Miltenyi Biotec OctoMACS tissue dissociator using the mouse lung protocol (m_lung_02). For iTreg cell sorting, the single-cell suspension was enriched using a CD4^+^ antibody (Miltenyi Biotec, catalog 130-101-962), and the lungs were stained using the reagents listed in Supplemental Table 5. A separate aliquot was taken for infiltrating immune cell analysis, which was enriched using CD45^+^ selection beads (Miltenyi Biotec, catalog 130-052-301), and the cells were stained using the reagents listed in Supplemental Table 5. Mice were included in the final analyses if their lungs displayed evidence of gross injury or they developed desaturation to at least 89%, indicating successful influenza infection.

For epithelial repair analysis after influenza-induced lung injury, the harvested post-caval lobes were injected with 2 mL Dispase (Corning, 354325) containing 0.25 mg DNase I per mL and incubated for 45 minutes at room temperature on a rocker. Afterward, forceps were used to tease apart large pieces of lung tissue followed by more thorough homogenization via vigorous pipetting through wide-bore pipette tips. The homogenized tissue was then incubated for another 10 minutes at room temperature on a rocker and then filtered through 40 μm filters. After centrifugation, the cells were resuspended in 2 mL ACK RBC lysis buffer and incubated for an additional 4 minutes at room temperature. The remaining cells were fixed and stained for flow cytometry analysis using the reagents listed in Supplemental Table 5. Data acquisition for analysis was performed using a Symphony A5 instrument with FACSDiva software (BD Biosciences). Analysis was performed with FlowJo software version 10.

For preparation of lungs for histopathology, mice were euthanized via carbon dioxide euthanasia chamber, and a tracheostomy was created. A 20-gauge angiocatheter was subsequently sutured into the trachea via the tracheostomy. The lungs and trachea were removed en bloc, and the lungs were inflated to 15 cm H_2_O with 4% paraformaldehyde. Five-micrometer sections from paraffin-embedded lungs were stained with H&E or trichrome and examined using light microscopy with a high-throughput, automated slide imaging system, TissueGnostics (TissueGnostics GmbH). The percentage of damaged lung area as a function of total lung area was determined using the image processing program ImageJ (NIH).

### Tissue preparation, flow cytometry, and cell sorting for in vitro RNA-Seq analysis.

For RNA-Seq analysis following in vitro culture, CD4^+^CD25^+^*Foxp3*-GFP^+^ Tregs or CD4^+^CD25^–^*Foxp3*-GFP^–^ T cells were isolated using the Miltenyi Biotec mouse CD4^+^CD25^+^ Regulatory T Cell Isolation Kit (catalog 130-091-041). For nTreg cell culture, 100,000 CD4^+^CD25^+^*Foxp3*-GFP^+^ Tregs were seeded in 96-well round-bottom plates (Corning, 3799) with αCD3ε/αCD28 Dynabeads (Gibco, 11456D) at a ratio of 3 beads to 1 nTreg and recombinant human IL-2 at a concentration of 2,000 U/mL (National Cancer Institute Frederick National Laboratory, Frederick, Maryland, USA). To generate iTregs, 300,000 CD4^+^CD25^–^*Foxp3*-GFP^–^ T cells were cultured in αCD3ε/αCD28–coated plates with recombinant human IL-2 (50 U/mL) and TGF-β (10 ng/mL). To delete UHRF1 at different times relative to FOXP3 induction, both cell types were cultured in tamoxifen at a concentration of 500 nM from day 0 to day 5 of culture (“early” groups) or from day 6 to day 12 of culture (“delayed” groups). On day 5 or 12 of cultures, cells were stained with CD4 and viability dye, then sorted using a FACSAria 6-Laser Sorter (BD Biosciences) for RNA-Seq analysis (Supplemental Table 5).

### Suppression assays.

For suppression assays, we sorted either direct ex vivo *Uhrf1^+/+^* CD4^+^*Foxp3*-GFP^+^ nTregs or *Uhrf1^fl/fl^* and *Uhrf1^+/+^* CD4^+^*Foxp3*-GFP^+^ iTregs from day 5 of early tamoxifen-treated cultures (described above), then cocultured each group at varying ratios with freshly isolated CD4^+^*Foxp3*-GFP^–^ Tconv cells labeled with CellTrace Violet (Invitrogen, C34557) (40). Treg:CD4^+^ Tconv cell cocultures were activated using αCD3ε/αCD28 Dynabead particles at a ratio of 3 beads to 1 Treg. After 72 hours, cells were harvested and analyzed by flow cytometry using a BD LSRFortessa. The division index of each sample was calculated using the proliferation modeling function in FlowJo software version 10: % suppression = 100 – (division index/division index of responders alone) × 100.

### RNA sequencing and analysis.

Adoptively transferred iTregs isolated from the spleen post-influenza or from in vitro–cultured cells were harvested from splenic single-cell suspension or cell culture, respectively, flow cytometry sorted, and lysed immediately after sorting with QIAGEN RLT Plus (catalog 1053393) containing 1% 2-mercaptoethanol. Cells were then subjected to simultaneous RNA and DNA isolation using the QIAGEN AllPrep Micro Kit (catalog 80204). RNA library preparation was performed using the SMARTer Stranded Total RNA-Seq Kit version 2 (Takara Bio, catalog 634411) as previously described (30, 56, 57). Sequencing was performed on an Illumina NextSeq 2000 instrument as previously described (30). For rare adoptively transferred iTregs, cells were flow cytometry sorted after harvesting from lung single-cell suspensions and lysed immediately after sorting with 10× RNA lysis buffer containing 5% RNase inhibitor. Fifty percent of the sample was taken for DNA isolation using the QIAGEN AllPrep Micro Kit (catalog 80204). RNA-Seq libraries were then prepared using 100 pg total RNA from each sample following the SMART-Seq v4 Ultra Low Input RNA Kit (Takara Bio) user manual. The cDNA was amplified with 11 cycles of PCR. The Nextera XT DNA Library Preparation Kit (Illumina) was used to make cDNA libraries suitable for Illumina sequencing. Prepared libraries were pooled at 4 nM and sequenced on a NextSeq 2000 (Illumina) using 75-base read lengths in single-end mode.

RNA-Seq analysis was performed as previously described (53). After sequencing, raw binary base call (BCL) files were converted to FASTQ files using BCL Convert (version 3.10.5, Illumina). Adapter trimming, alignment to the GRCm38 reference genome, and quantification were performed using the nf-core/RNA-Seq pipeline version 3.9 (implemented in Nextflow 22.04.5 [https://nf-co.re/rnaseq] with Northwestern University Genomics Compute Cluster configuration [nextflow run nf- core/rnaseq -profile nu_genomics --genome GRCm38]). Differential expression analysis was performed in R package DEseq2 (version 1.38.3 in R 4.2.3). For *k*-means analysis, *k* was determined using elbow plots, and the *k*-means function in R stats 3.6.2 (Hartigan-Wong method with 25 random sets and a maximum of 1,000 iterations) was used for clustering. In vitro *k*-means heatmaps were generated using the Morpheus web interface (https://software.broadinstitute.org/morpheus/). Gene set enrichment analysis (GSEA) was performed using the Broad Institute’s GSEA software, version 4.1.0, GSEAPreranked tool (58) with genes ordered by log_2_(fold change) in average expression against the hallmark gene sets or the immunologic signature gene sets housed in the Molecular Signatures Database of the Broad Institute (59).

### Modified reduced-representation bisulfite sequencing and reduced-representation enzymatic methylation sequencing and analysis.

Modified reduced-representation bisulfite sequencing (mRRBS) library preparation for in vitro–cultured cells was performed using procedures previously described by our group (30, 40, 60). Briefly, genomic DNA was isolated from sorted samples via the QIAGEN AllPrep Micro Kit (catalog 80204) and quantified with a Qubit 3.0 instrument (Invitrogen). Bisulfite conversion was then performed using the EZ DNA Methylation-Lightning Kit (Zymo Research) per the manufacturer’s protocol. We next created indexed Illumina-compatible non-directional libraries from bisulfite-converted single-stranded DNA using the Pico Methyl-Seq Library Prep Kit (Zymo Research). Final library size distribution and quality were assessed via high-sensitivity screen tape (TapeStation 4200, Agilent). Libraries were then pooled for sequencing on a NextSeq 2000 (Illumina) instrument using the V2 High Output reagent kit (1 × 75 cycles).

Reduced-representation enzymatic methylation sequencing (RREM-seq) library preparation was performed as previously described (61, 62). Briefly, after genomic DNA extraction using the QIAGEN AllPrep DNA/RNA Micro Kit (catalog 80204), 0.5–1 ng of genomic DNA was digested using restriction endonuclease MspI (New England Biolabs) and then enzymatically converted with TET2 and APOBEC (New England Biolabs) per the manufacturer’s instructions. Random priming, adapter ligation, PCR product clean-up, and final library amplification were performed using the Pico Methyl-Seq Library Prep Kit (Zymo Research). Unmethylated λ-bacteriophage DNA (1:200 mass ratio; New England Biolabs) was included in all samples to calculate unmethylated cytosine conversion efficiency (on average >99%). Final library size distribution and quality were assessed via high-sensitivity screen tape (TapeStation 4200, Agilent) and sequenced using single-end reads with a NextSeq 2000 (Illumina).

Methylation analysis was conducted as previously described (30, 61). Briefly, after sequencing, raw BCL files were converted to FASTQ files using BCL Convert (version 3.10.5, Illumina) and trimmed using Trim Galore! (version 0.4.3) (Babraham Institute). Bismark (version 0.21.0) (Babraham Institute) was used to perform alignment to the reference genome mm10 (GRCm38) and methylation extraction. Bismark coverage files were used for quantification using SeqMonk (version 1.48.0) (Babraham Institute) and R package DSS (version 2.46.0). Cumulative distribution plots were generated with the ecdf base R function.

### Statistics.

*P* values and *q* values resulting from 2-tailed tests were calculated using statistical tests stated in the figure legends. Statistical analysis was performed using either GraphPad Prism version 10.3.0 or R version 4.2.3. A *P* or *q* value of less than 0.05 was considered significant, except for GSEA, in which 0.25 was considered significant (58). Outliers were identified and removed via the ROUT method at a *Q* of 5%. Computational analysis was performed using Genomics Nodes and Analytics Nodes on Quest, Northwestern University’s High-Performance Computing Cluster.

### Study approval.

All animal experiments and procedures were conducted in accordance with the standards established by the US Animal Welfare Act set forth in NIH guidelines and were approved by the IACUC at Northwestern University under protocols IS00012519 and IS00017837.

### Data availability.

The raw sequencing data are available in the NCBI’s Gene Expression Omnibus (GEO) repository under accession number GSE290605. Values for all data points in graphs are reported in the Supporting Data Values file.

## Author contributions

AMJ, JKG, MATA, SEW, EMS, and BDS contributed to the conception, hypothesis delineation, and design of the study. AMJ, JKG, QL, MATA, KAH, LMN, NM, CPRF, HAV, EMS, SEW, and BDS performed experiments/data acquisition and analysis. AMJ, EMS, and BDS wrote the manuscript or provided substantial involvement in its revision.

## Funding support

This work is the result of NIH funding, in whole or in part, and is subject to the NIH Public Access Policy. Through acceptance of this federal funding, the NIH has been given a right to make the work publicly available in PubMed Central.

- NIH awards F32HL162418 (to AMJ); T32GM144295, T32HL076139, and F31HL162490 (to MATA); T32AI083216 (to NM); T32HL076139 (to CPRF); T32HL076139 (to JKG); K08HL159356 and U19AI135964 (to LMN); and R01HL149883, R01HL153122, P01HL154998, P01AG049665, U19AI135964, and U19AI181102 (to BDS).
- The David W. Cugell Fellowship (to QL).
- Genomics Network (GeNe) Pilot Project Funding (to QL).
- The Parker B. Francis Opportunity Award (to LMN).
- Burroughs Wellcome Fund Career Award for Medical Scientists (to SEW).
- NIH CA060553 supported the Northwestern University Flow Cytometry Core Facility; NIH S10OD011996 supported the purchase of the BD FACSAria SORP system.
- NIH P30CA060553 awarded to the Robert H. Lurie Comprehensive Cancer Center supported the Northwestern University Mouse Histology and Phenotyping Laboratory.

## Supplementary Material

Supplemental data

Supplemental table 1

Supplemental table 2

Supplemental table 3

Supplemental table 4

Supporting data values

## Version 1 09/16/2025

In-Press Preview

## Version 2 11/17/2025

Electronic publication
