# Supplementary material for: Maintenance DNA methylation is required for induced Treg reparative function following viral pneumonia in mice
Source: J Clin Invest. 2025 Sep 16;135(22):e192925. doi: 10.1172/JCI192925 (PMC12618063; doi:10.1172/JCI192925)
Supplement: Supplemental data [file jci-135-192925-s276.pdf]

Supplement

Maintenance DNA methylation is required for induced Treg reparative function following viral pneumonia in mice

Supplemental Table Legends

Supplemental Table 1. DEG lists used to generate Figures 2E-F, 5B-C, 6D, Supplemental 3A, Supplemental 7B-E. Provided as a multi-tabbed Excel file.

Supplemental Table 2. Ranked gene list used to generate Figure 2G and Supplemental Figure 3D (day 12 "delayed" *Uhrf1*$^{+/+}$ versus day 12 "delayed" *Uhrf1*$^{fl/fl}$ iTregs) against a comprehensive list of 4,872 Immunologic Signature gene sets (Figure 2G) and 50 Hallmark gene sets (Supplemental Figure 3D) housed in the Molecular Signatures Database. [provided as a tab-delimited file]

Supplemental Table 3. Ranked gene list used to generate Figures 5D-E (day 24 post influenza *Uhrf1*$^{+/+}$ versus *Uhrf1*$^{fl/fl}$ iTregs) against a comprehensive list of 50 Hallmark gene sets (Figure 5D-E) and 3,930 GO Biological Process gene sets (Figure 5F) housed in the Molecular Signatures Database. [provided as a tab-delimited file]

Supplemental Table 4. Ranked gene list used to generate Figures 6B-C (day 11 post influenza *Uhrf1*$^{+/+}$ versus *Uhrf1*$^{fl/fl}$ iTregs) against a comprehensive list of 50 Hallmark gene sets (Figure 6B) and 3,173 GO Biological Process gene sets (Figure 6C) housed in the Molecular Signatures Database. [provided as a tab-delimited file]

Supplemental Table 5. Flow cytometry fluorochromes and reagents.

Antigen/Reagent	Conjugate	Clone	Manufacturer	Catalog no.
CD4	APC-eFluor™ 780	RM4-5	Invitrogen	47-0042-82
DAPI/Cell-Impermeant Dye	N/A	N/A	Thermo Fisher Scientific	62248
CD45	FITC	30-F11	BioLegend	103107
CD31	PE	MEC 13.3	BD Pharmingen	553373
Podoplanin	PE-Cyanine7	eBio8.1.1	eBioscience	25-5381-82
CD326	BV421	G8.8	BioLegend	118225
Krt5<br>Krt5 Secondary Antibody	Unconjugated<br>Alexa Fluor 488	Poly9059	BioLegend<br>Invitrogen	905901<br>A11039
MHCII	BUV395	2G9	BD OptiBuild	743876
Ki-67	PerCP-eFluor 710	SolA15	eBioscience	46-5698-82
Fixable Viability Dye eFluor 506	N/A	N/A	eBioscience	65-0866-14
CD45	APC-Cy7	30-F11	BD Pharmingen	557659
CD3ε	FITC	145-2C11	Invitrogen	11-0031-85

CD4	BUV395	GK1.5	BD Horizon	565974
CD8	PECF594	53-6.7	BioLegend	100762
CD64	PE	X54-5/7.1	BioLegend	139304
CD11b	BUV737	M1/70	BD Horizon	612800
CD11c	Pe-Cy7	HL3	BD Pharmingen	558079
SiglecF	AF647 (APC)	E50-2440	BD Pharmingen	562680
Ly6G	AF 700 (A700)	1A8	BD Pharmingen	561236
CountBright™ Absolute Counting Beads	N/A	N/A	Invitrogen	C36950

Supplemental Figure 1

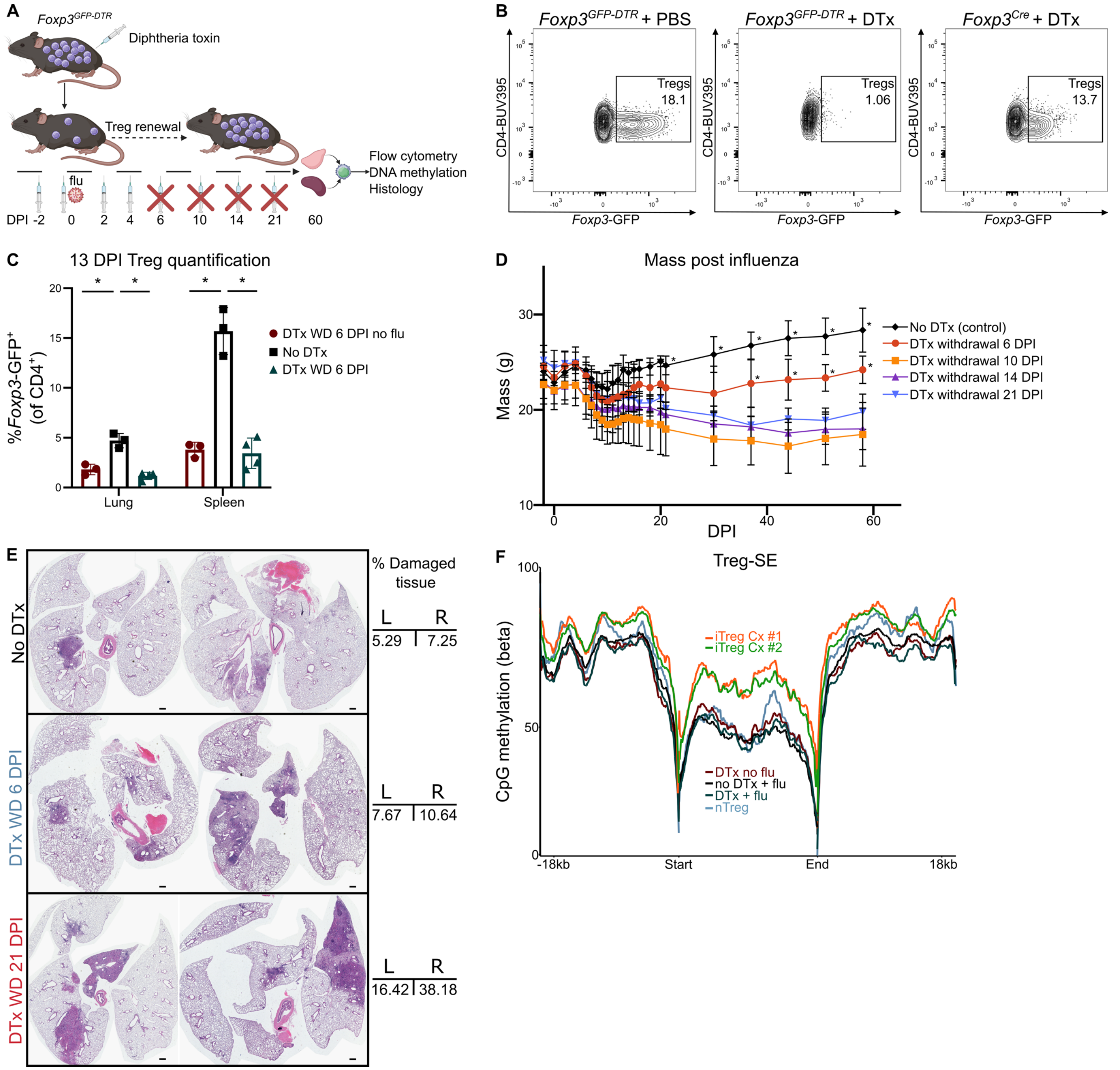

Supplemental Figure 1: *Effect of Treg renewal on recovery following influenza A virus infection.*

Foxp3$^{GFP\text{-}DTR}$ mice treated with DTx every 48 hours beginning two days prior to inoculation with 5 PFUs of influenza A/WSN/33 H1N1 virus. DTx was subsequently withdrawn (WD) on pre-specified DPIs (6, 10, 14, 21). **(A)** Schematic. **(B)** Representative flow plots of endogenous splenic CD4^{+}*Foxp3*-GFP^{+} Treg population depletion in *Foxp3*$^{GFP\text{-}DTR}$ mice following the administration of four doses of DTx (6 DPI). *Foxp3*$^{GFP\text{-}DTR}$ and *Foxp3*^{Cre} mice that received PBS and DTx, respectively,

included for comparison. **(C)** Lung and spleen *Foxp3*-GFP$^+$ Treg cell quantification in mice on 13 DPI that had DTx withdrawn on 6 DPI, (DTx WD 6 DPI, n=4), compared with mice that did not receive DTx (No DTx, n=3), or had DTx withdrawn on 6 DPI but did not receive influenza (DTx WD 6 DPI no flu n= 3). **(D)** Mass of mice described in Supplemental Figure 1A (n=4 for DTx WD 6 DPI group, n=5 for all other groups). **(E)** Representative lung histopathology (H&E staining) and percent damaged tissue at 60 DPI of control (No DTx) and DTx withdrawal (WD) mice. Original magnification x10, scale bar = 1 mm. **(F)** Metagene analysis of DNA methylation across the Treg-SE of Tregs recovered on 13 DPI from mice described in Supplemental Figure 1C compared with naïve splenic nTregs and two biological culture replicates of iTregs harvested on day 5 of culture with IL-2 and TGF-β . Independent biological replicates are shown. Data presented as mean and SD. * $q < 0.05$ according to one-way ANOVA with two-stage linear step-up procedure of Benjamini, Krieger, and Yekutieli with Q = 5% **(C)**.* $q < 0.05$ according to mixed-effects model (REML) with two-stage linear step-up procedure of Benjamini, Krieger, and Yekutieli with Q = 5% **(D)**. Data in **C-F** are each from 1 independent experiment.

Supplemental Figure 2

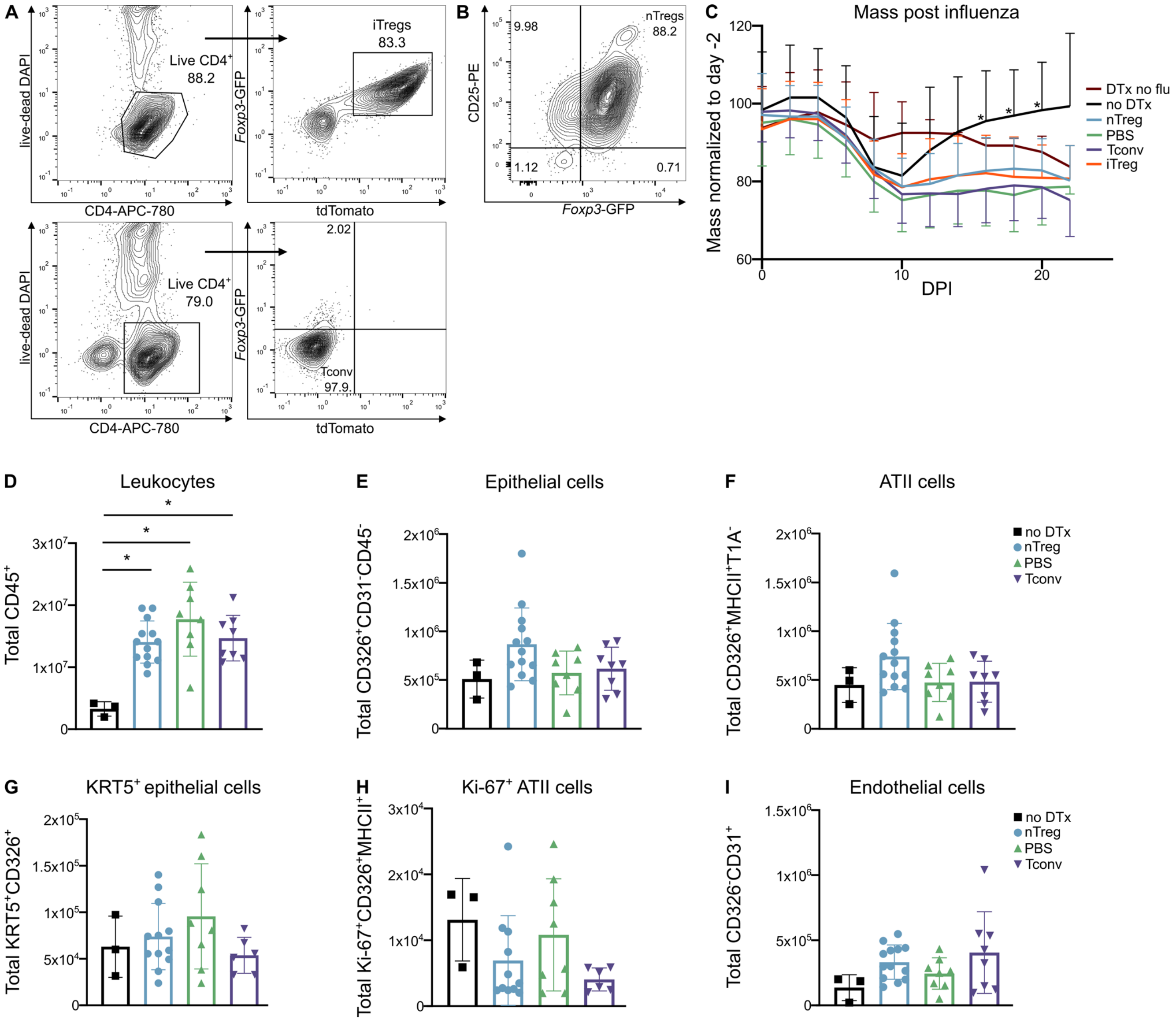

Supplemental Figure 2: *Effects of receiving DTx or no DTx.*

(A) Flow cytometry contour plots phenotyping CD4$^+$*Foxp3*-GFP$^+$tdTomato$^+$ iTreg and CD4$^+$*Foxp3*-GFP$^-$tdTomato$^-$ conventional T cell populations following 3 days of culture in the presence of αCD3ε/αCD28, IL-2, TGF-β, and tamoxifen (iTregs) or αCD3ε/αCD28 and IL-2 (Tconv). **(B)** Flow cytometry contour plot of CD4$^+$CD25$^+$*Foxp3*-GFP$^+$ nTregs isolated from the spleens of mice prior to adoptive transfer. **(C)** Mass over time of *Foxp3*$^{GFP\text{-}DTR}$ mice treated with DTx every 48 hours beginning 2 days prior to intra-tracheal inoculation of 6.5 PFU of influenza A/WSN/33 H1N1 virus, and adoptive transfer of nTreg (n=27), PBS (n=21), Tconv (n=25), or iTreg (n=18) cells on 5 DPI. Positive controls included mice that received

influenza but no DTx (no DTx, n=9) and DTx but no influenza (DTx no flu, n=3). **(D-I)** Mice were euthanized on 24 DPI and lungs were analyzed by flow cytometry for total **(D)** $CD45^+$ cells, **(E)** $CD326^+CD31^-CD45^-$ epithelial cells, **(F)** $CD326^+MHCII^+T1A^-$ ATII cells, **(G)** $KRT5^+CD326^+$ epithelial cells, **(H)** Ki-$67^+CD326^+MHCII^+$ ATII cells, and **(I)** $CD326^-CD31^+$ endothelial cells. (**D-F**, No DTx n=3, nTreg n=13, PBS n=8, Tconv n=8; **G**, No DTx n=3, nTreg n=12, PBS n=8, Tconv n=6; **H**, No DTx n=3, nTreg n=11, PBS n=8, Tconv n=6; **I**, No DTx n=3, nTreg n=13, PBS n=8, Tconv n=8). * $q < 0.05$ according to mixed-effects model (REML) with two-stage linear step-up procedure of Benjamini, Krieger, and Yekutieli with Q = 5% **(C)**. Data presented as mean and SD **(D-I)** with * $q < 0.05$ according to multiple Mann-Whitney tests and correcting for multiple comparisons using the two-stage linear step-up procedure of Benjamini, Krieger, and Yekutieli with Q = 5% **(D)**. Corresponding total cell numbers from recipients of iTregs not shown as analysis of those populations was derived from the post-caval lobe, as opposed to whole lung suspensions. Data in **C** generated from five independent experiments. Data in **D-I** generated from two independent experiments.

Supplemental Figure 3

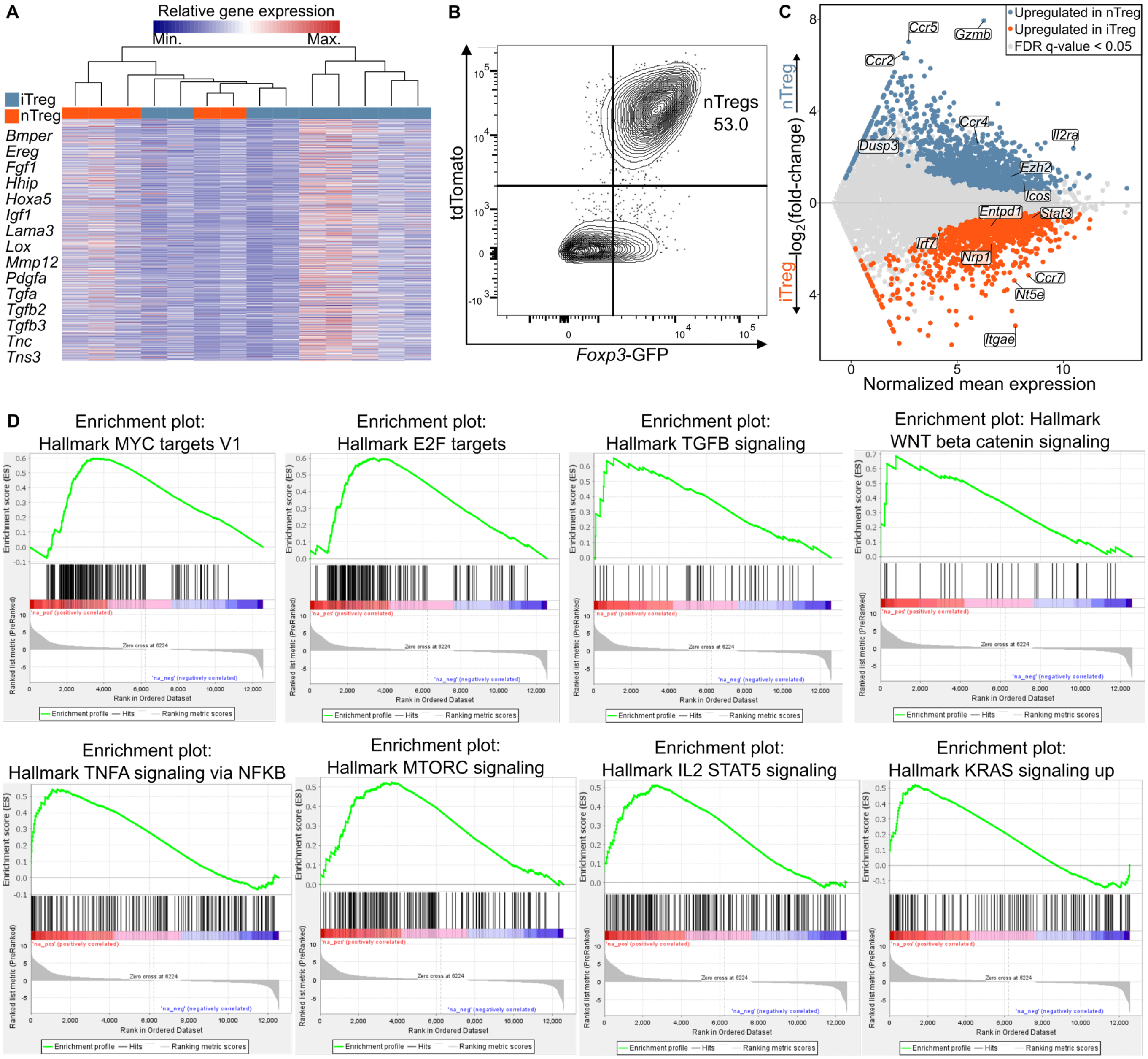

Supplemental Figure 3: *Confirmation of canonical Treg transcriptomic signature in nTregs and iTregs.*

(A) Heatmap comparing normalized counts of genes previously identified to be enriched and associated with repair function in nTregs harvested from the lungs of 8-12 week-old influenza infected *Foxp3*$^{GFP\text{-}DTR}$ mice at 60 DPI with adoptively transferred *Uhrf1*$^{+/+}$ iTregs at 24 DPI. Gene list derived from cluster II of K-means heat map shown in Figure 4 (40). Genes of interest are annotated. **(B)** Representative flow cytometry contour plot analysis of nTreg cells on day 5 of culture with tamoxifen. **(C)** MA plot comparing gene expression of *Uhrf1*$^{+/+}$ iTregs (control) with *Uhrf1*$^{+/+}$CD4$^+$*Foxp3*-GFP$^+$ nTregs on day

5 of culture. Induced Tregs were generated via culture of $CD4^+$*Foxp3*-GFP^- T cells in the presence of αCD3ε/αCD28 coated to the plate, recombinant human IL-2 at a concentration of 50 U/ml, and TGF-β at a concentration of 10ng/ml. Natural Tregs were cultured in the presence of recombinant human IL-2 at a concentration of 2,000 U/ml and αCD3ε/αCD28 activation beads at a ratio of three beads to one Treg cell as in Figure 2A. Genes of interest are annotated. **(D)** Enrichment plots of gene sets generated through GSEA pre-ranked testing of the expressed genes of delayed *Uhrf1*$^{+/+}$ iTregs (control) and delayed *Uhrf1*$^{fl/fl}$ iTregs on day 12 of culture.

Supplemental Figure 4

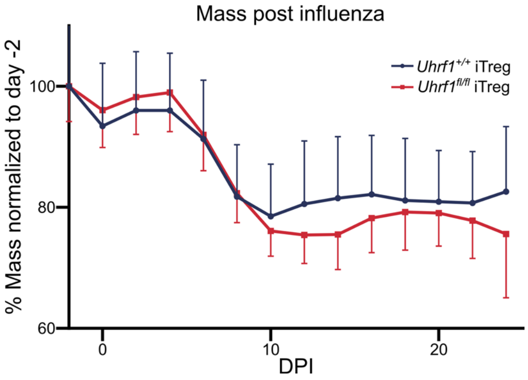

Supplemental Figure 4: iTreg *UHRF1 is dispensable for promoting mass recovery following viral pneumonia.*

Mass over time of recipient *Foxp3*$^{GFP\text{-}DTR}$ mice that were treated with DTx every 48 hours beginning two days before inoculation with 6.5 PFUs of influenza A/WSN/33 H1N1 virus and then received retroorbital adoptive transfer of 1x10^6 *Foxp3*-GFP$^+$tdTomato$^+$ *Uhrf1*$^{fl/fl}$ (n=15) or *Uhrf1*$^{+/+}$ (n=18) iTregs on 5 DPI as in Figure 1. iTregs were treated with tamoxifen from culture day 0 to day 3 and were harvested for adoptive transfer on culture day 5. Data generated from four independent experiments.

Supplemental Figure 5

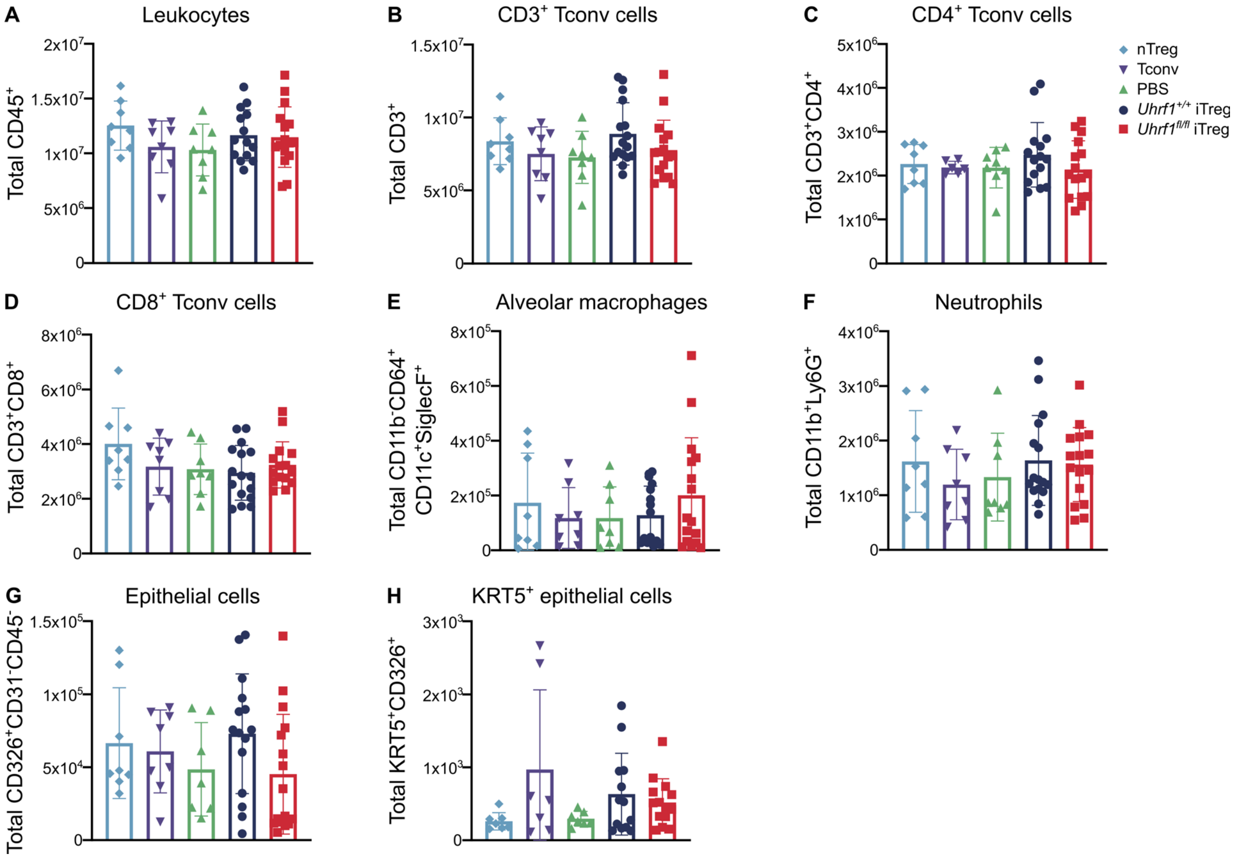

Supplemental Figure 5: *UHRF1 is dispensable for iTreg suppression of infiltrating immune cells during viral pneumonia.*

Foxp3$^{GFP\text{-}DTR}$ mice were treated with DTx every 48 hours beginning 2 days prior to inoculation with 6.5 PFUs of influenza A/WSN/33 H1N1 virus, and then received retroorbital adoptive transfer of 1x10^6 nTregs, Tconv, *Uhrf1*$^{fl/fl}$ *iTregs, Uhrf1*$^{+/+}$ iTregs, or PBS on 5 DPI. iTregs were treated with tamoxifen from culture day 0-3 and then harvested for transfer on day 5. Natural Tregs were adoptively transferred directly following isolation from the spleens and lymph nodes of mice. Recipients were euthanized on 11 DPI and lungs were analyzed by flow cytometry for total **(A)** CD45$^+$, **(B)** CD3$^+$, **(C)** CD4$^+$CD8$^-$, **(D)** CD4$^-$CD8$^+$, **(E)** CD11b$^-$CD64$^+$CD11c$^+$SiglecF$^+$, **(F)** CD11b$^+$Ly6G$^+$, **(G)** CD326$^+$CD31$^-$CD45$^-$ (epithelial), or **(H)** KRT5$^+$CD326$^+$ epithelial cells. Data in **G** and **H** derived exclusively from the post caval lobes. (**A**, nTreg (n=8), Tconv (n=8), PBS (n=8), *Uhrf1*$^{+/+}$ iTregs (n=14), U*hrf1*$^{fl/fl}$ iTregs (n=16); **B**, nTreg (n=8), Tconv (n=8), PBS (n=8), *Uhrf1*$^{+/+}$ iTregs (n=16), U*hrf1*$^{fl/fl}$ iTregs (n=16); **C**, nTreg (n=8), Tconv (n=6), PBS (n=8), *Uhrf1*$^{+/+}$ iTregs (n=15), U*hrf1*$^{fl/fl}$ iTregs (n=16); **D-E**, nTreg (n=8), Tconv (n=8), PBS (n=8), *Uhrf1*$^{+/+}$ iTregs (n=16), U*hrf1*$^{fl/fl}$ iTregs (n=15); **F**, nTreg (n=8), Tconv (n=8), PBS (n=8), *Uhrf1*$^{+/+}$ iTregs (n=16), U*hrf1*$^{fl/fl}$ iTregs (n=16); **G**, nTreg (n=8), Tconv (n=8), PBS (n=7), *Uhrf1*$^{+/+}$ iTregs (n=16), U*hrf1*$^{fl/fl}$ iTregs (n=16);

H, nTreg (n=7), Tconv (n=7), PBS (n=7), *Uhrf1*$^{+/+}$ iTregs (n=14), U*hrf1*$^{fl/fl}$ iTregs (n=14). Data presented as mean and SD. Not significant by multiple Mann-Whitney tests and correcting for multiple comparisons using the two-stage linear step-up procedure of Benjamini, Krieger, and Yekutieli with Q = 5% test. Data generated from two independent experiments.

Supplemental Figure 6

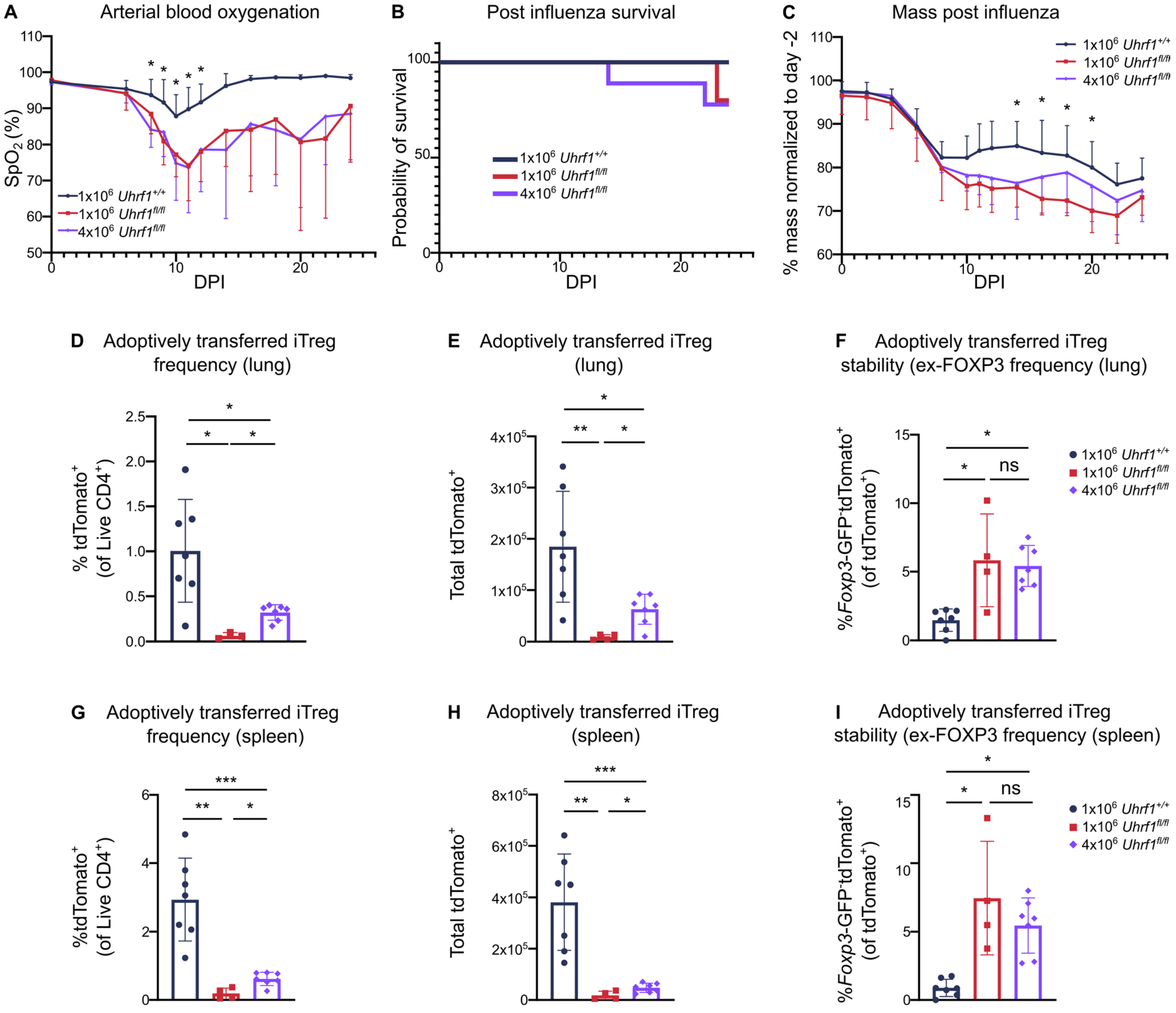

Supplemental Figure 6: Greater magnitude of *Uhrf1*$^{fl/fl}$ iTreg adoptive transfer fails to equalize tissue engraftment with *Uhrf1*$^{+/+}$ iTregs during viral pneumonia.

Foxp3$^{GFP\text{-}DTR}$ mice treated with DTx every 48 hours beginning 2 days before inoculation with 6.5 PFUs of influenza A/WSN/33 H1N1 virus, then received adoptive transfer of 1x10^{6} *Uhrf1*$^{+/+}$ iTregs, 1x10^{6} *Uhrf1*$^{fl/fl}$ iTregs, or 4x10^{6} *Uhrf1*$^{fl/fl}$ iTregs on 5 DPI. iTregs were treated with tamoxifen from culture day 0-3, and harvested for adoptive transfer on culture day 5. Surviving mice were euthanized on 24 DPI and lungs and spleens were analyzed by flow cytometry. **(A-C)** *Foxp3*$^{GFP\text{-}DTR}$ mice that received 1x10^{6} *Uhrf1*$^{+/+}$ iTregs (n=7), 1x10^{6} *Uhrf1*$^{fl/fl}$ iTregs (n=5), or 4x10^{6} *Uhrf1*$^{fl/fl}$ iTregs (n=9) were measured for **(A)** arterial oxyhemoglobin saturation (SpO_2), **(B)** survival, or **(C)** mass. **(D-F)** Lungs from 1x10^{6} *Uhrf1*$^{+/+}$ iTregs, 1x10^{6}

Uhrf1$^{fl/fl}$ iTregs, or 4x10^{6} *Uhrf1*$^{fl/fl}$ iTreg recipients were isolated and analyzed for (**D)** frequency of tdTomato^{+} cells (1x10^{6} *Uhrf1*$^{+/+}$ iTregs n=7*;* 1x10^{6} *Uhrf1*$^{fl/fl}$ iTregs n=3; or 4x10^{6} *Uhrf1*$^{fl/fl}$ iTregs n=7), **(E)** total number of tdTomato^{+} cells (1x10^{6} *Uhrf1*$^{+/+}$ iTregs n=7; 1x10^{6} *Uhrf1*$^{fl/fl}$ iTregs n=4; or 4x10^{6} *Uhrf1*$^{fl/fl}$ iTregs n=7) and (**F)** frequency of Foxp3^{-}tdTomato^{+} (ex-FOXP3) cells (1x10^{6} *Uhrf1*$^{+/+}$ iTregs n=7; 1x10^{6} *Uhrf1*$^{fl/fl}$ iTregs n=4; or 4x10^{6} *Uhrf1*$^{fl/fl}$ iTregs n=7). **(G-I)** Spleens from 1x10^{6} *Uhrf1*$^{+/+}$ iTregs (n=7), 1x10^{6} *Uhrf1*$^{fl/fl}$ iTregs (n=4), or 4x10^{6} *Uhrf1*$^{fl/fl}$ iTregs (n=7) recipients were isolated and analyzed for **(G)** frequency of tdTomato^{+} cells, **(H)** total number of tdTomato^{+} cells, and **(I)** frequency of Foxp3^{-}tdTomato^{+} (ex-FOXP3) cells. Data presented as mean and SD with * $p < 0.05$, **$p < 0.005$, according to multiple Mann-Whitney U tests and correcting for multiple comparisons using the two-stage linear step-up procedure of Benjamini, Krieger, and Yekutieli with Q = 5%, ns, not significant **(D-I)**. * $q < 0.05$ according to mixed-effects model (REML) with two-stage linear step-up procedure of Benjamini, Krieger, and Yekutieli with Q = 5% **(A, C)**. Data in **A-I** generated from one independent experiment.

Supplemental Figure 7

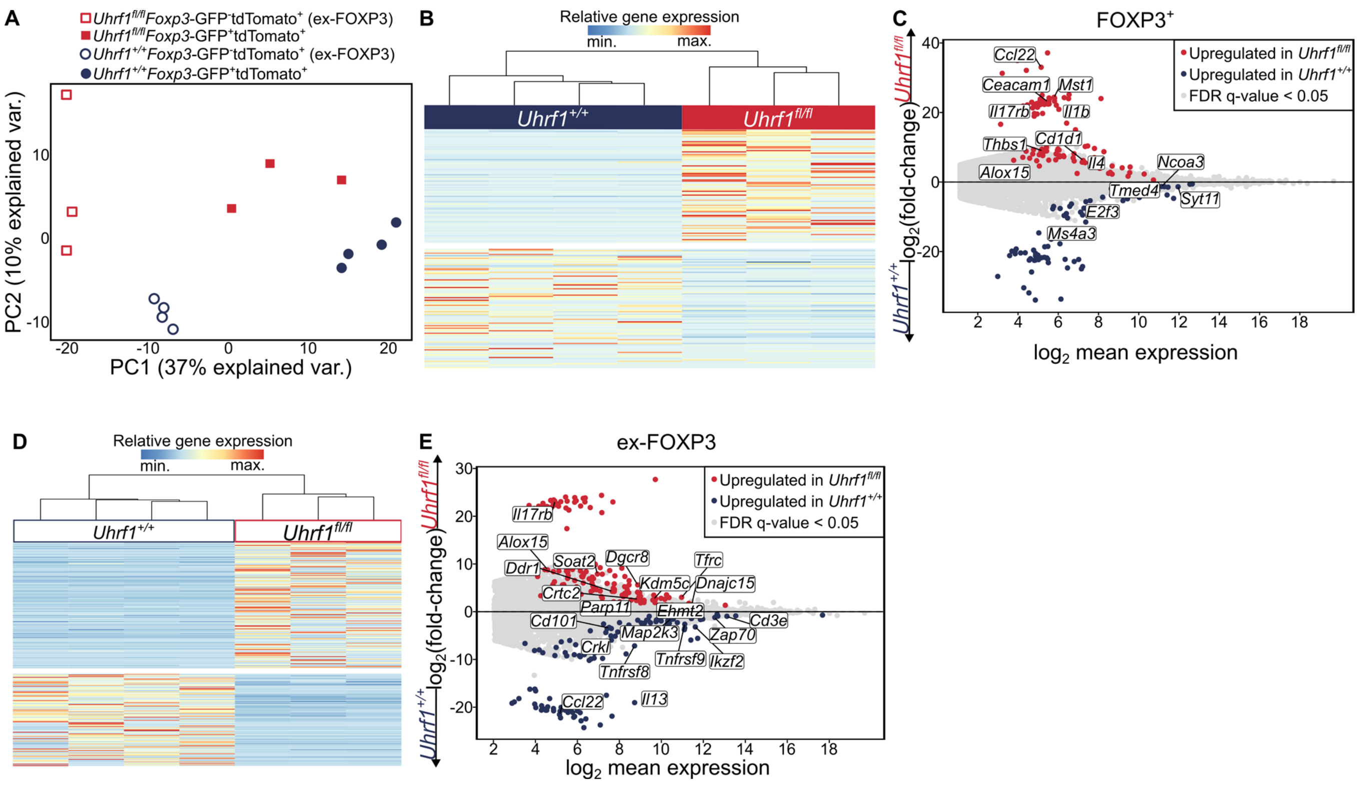

Supplemental Figure 7: *UHRF1 is required for iTreg phenotypic stability following viral pneumonia.*

Foxp3$^{GFP\text{-}DTR}$ mice treated with DTx every 48 hours beginning 2 days before inoculation with 6.5 PFUs of influenza A/WSN/33 H1N1 virus, then received adoptive transfer of 1x10^{6} *Uhrf1*$^{+/+}$ iTregs (n=4) or 1x10^{6} *Uhrf1*$^{fl/fl}$ iTregs (n=3) on 5 DPI. iTregs were treated with tamoxifen from culture day 0-3 and then harvested for adoptive transfer on culture day 5. On day 24 post infection *Foxp3*-GFP^{+}tdTomato^{+} and *Foxp3*-GFP^{-}tdTomato^{+} (ex-FOXP3) *Uhrf1*$^{+/+}$ or *Uhrf1*$^{fl/fl}$ cells were sorted from single-cell suspensions of spleens for quantification and gene expression profiling. **(A)** PCA of 457 differential expressed genes from sorted *Uhrf1*$^{+/+}$ and *Uhrf1*$^{fl/fl}$ cells harvested from the spleen 24 DPI identified from ANOVA-like testing with FDR $q < 0.05$. **(B)** K-means clustering of 183 genes with FDR $q < 0.05$ comparing FOXP3^{+} *Uhrf1*$^{+/+}$ and *Uhrf1*$^{fl/fl}$ iTregs harvested from the spleens of *Foxp3*$^{GFP\text{-}DTR}$ mice at 24 DPI with k = 2. **(C)** MA plot comparing gene expression of adoptively transferred FOXP3^{+} *Uhrf1*$^{+/+}$ and *Uhrf1*$^{fl/fl}$ iTregs harvested from the spleen 24 DPI. Genes of interest are annotated. **(D)** K-means clustering of 274 genes with FDR $q < 0.05$ comparing ex-FOXP3 *Uhrf1*$^{+/+}$ and *Uhrf1*$^{fl/fl}$ cells harvested from the spleens of *Foxp3*$^{GFP\text{-}DTR}$ mice at 24 DPI with k = 2. **(E)** MA plot comparing gene expression of adoptively

transferred ex-FOXP3 *Uhrf1*$^{+/+}$ and *Uhrf1*$^{fl/fl}$ cells harvested from the spleen 24 DPI. Genes of interest are annotated. Data in **A-E** generated from 1 independent experiment. Independent biological replicates are shown.

Supplemental Figure 8

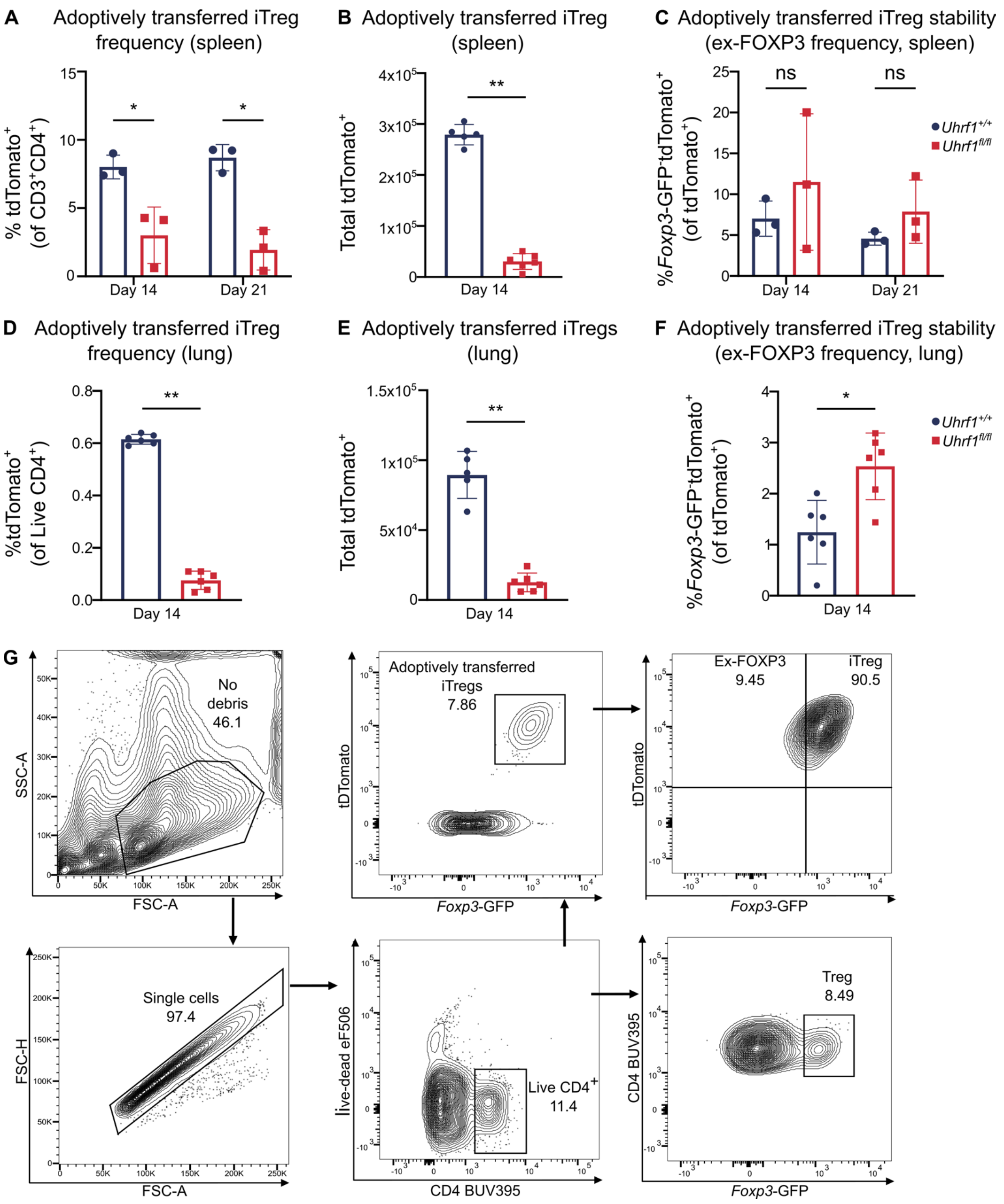

Supplemental Figure 8: *UHRF1 is required for iTreg lung and splenic tissue engraftment in the absence of viral pneumonia.*

Foxp3$^{GFP-DTR}$ mice were treated with DTx every 48 hours starting on day 0 and received retroorbital adoptive transfer of 1x10^{6} *Uhrf1$^{+/+}$* iTregs or 1x10^{6} *Uhrf1$^{fl/fl}$* iTregs on day 5. iTregs were treated with tamoxifen from culture day 0-3 and then were harvested for adoptive transfer on culture day 5. Mice were euthanized on day 14 or 21 and the spleens and lungs were analyzed by flow cytometry. **(A)** Frequency of tdTomato^{+} cells recovered from the spleens at day 14 and day 21 of recipients of *Uhrf1$^{fl/fl}$* or *iUhrf1$^{+/+}$* iTregs (n=3 per group). **(B)** Total tdTomato^{+} cells recovered from the spleen at day 14

from recipients of *Uhrf1*$^{fl/f}$ (n=6) or *iUhrf1*$^{+/+}$ iTregs (n=5). **(C)** Frequency of *Foxp3*-GFP^{-}tdTomato^{+} (ex-FOXP3) cells recovered at day 14 and day 21 from the spleens of recipients of *Uhrf1*$^{fl/fl}$ or *Uhrf1*$^{+/+}$ iTregs (n=3 per group). **(D)** Frequency of tdTomato^{+} cells recovered from the lungs at day 14 of recipients of *Uhrf1*$^{fl/fl}$ or *iUhrf1*$^{+/+}$ iTregs (n=6 per group). **(E)** Total number of tdTomato^{+} cells recovered from the lungs at day 14 of recipients of *Uhrf1*$^{fl/fl}$ (n=6) or *iUhrf1*$^{+/+}$ iTregs (n=5). **(F)** Frequency of *Foxp3*-GFP^{-}tdTomato^{+} (ex-FOXP3) cells recovered at day 14 from the lungs of recipients of *Uhrf1*$^{fl/fl}$ or *Uhrf1*$^{+/+}$ iTregs (n=6 per group). **(G)** Representative gating strategy of splenic Ex-FOXP3 population presented in Supplemental Figure 8C. Data presented as mean and SD with * p < 0.05 according to Mann-Whitney U test. Data in **A and C** are representative of 2 independent experiments. Data in **B and D-F** generated from 1 independent experiment.
